# Toward Energy-Efficient and Low-Carbon Intrusion Detection in Edge and Cloud Computing Based on GreenShield Cybersecurity Framework

**DOI:** 10.3390/s26061780

**Published:** 2026-03-11

**Authors:** Abdullah Alshammari

**Affiliations:** College of Computer Science and Engineering, University of Hafr Albatin, Hafar Albatin 31991, Saudi Arabia; dr.abdullah@uhb.edu.sa or alshammari@ieee.org

**Keywords:** green cybersecurity, energy-efficient intrusion detection, sustainable edge computing, carbon-aware cloud security, lightweight cryptographic protocols, federated learning, knowledge distillation

## Abstract

The fast growth of edge–cloud computing infrastructures has increased the cybersecurity burden even as it has substantially amplified the energy use and carbon footprint of intrusion detection systems (IDSs). In order to overcome this challenge, this paper suggests GreenShield, which is a framework of low-carbon cybersecurity involving lightweight cryptography, deep learning that is energy efficient, and carbon conscious system optimization across distributed edges and in cloud setup. GreenShield employs a hierarchical federated learning architecture with integrated knowledge distillation and a carbon-aware scheduling controller that dynamically adjusts security response execution based on threat intensity and renewable energy availability. As extensive experiments on the UNSW-NB15 and CIC-IDS2017 datasets show, GreenShield attains 98.73% detection accuracy and is 67.4% more energy efficient than traditional deeplearning-based IDSs. Further, the suggested system reduces the operational carbon emissions up to 97.6%, which is equivalent to a reduction of around 2.8 kg CO2-equivalent/per hour in a typical edge-deployment situation, yet it does not undermine the performance of the detection. These findings suggest that GreenShield can be one of the meaningful alternatives in providing viable and scalable sustainable cybersecurity that supports carbon-conscious security workflows in the future edge–cloud computing architecture.

## 1. Introduction

The emerging boom in edge and cloud computing systems has indeed changed the landscape of the modern digital ecosystems, giving unexplainable connectivity and computing capabilities in many areas of implementation distinctions, including smart cities, autonomous automobiles, industrial automation and healthcare systems [1,2,3]. However, there are also certain harsh challenges that this change has initiated the cybersecurity and the environmental sustainability nexus. The data patterns at present are consuming about 1–1.5% of total electricity in the world and there are projections that it may increase further to 8% by 2030 [4,5,6]. Security operations, especially intrusion detection systems, and cryptographic procedures, take up a large part of this energy consumption, which needs new methods that would be both protection-acceptable and eco-friendly [7,8,9,10].

Traditional cybersecurity systems were not engineered to be energy efficient or to produce any carbon footprint, the emphasis was on the accuracy with which it detected as well as the speed of its response, rather than the computational sustainability [11,12,13]. Deep learning-enabled intrusion detection systems, although proving to be more performance effective in detecting advanced attacks, generally consume a lot of computed power to generate a substantial amount of energy use and carbon footprint [14,15,16,17,18]. As an example, training a common convolutional neural network on the classification of network traffic may use the energy that would be consumed by five cars throughout their lifespan [19,20,21,22]. This paradigm is becoming unsustainable because organizations are under increasing pressure due to the pressure imposed on them by the regulatory frameworks and the stakeholder expectations coupled with real environmental issues that require the organizations to minimize their carbon footprint [23,24,25,26].

Sustainable implementation of cybersecurity in edge computing environments has its own peculiarities. Edge devices with a limited number of resources need to implement security functions with low power limits and low latency reaction to the possible threats [27,28,29,30]. The distributed form of edge architectures further complicates the optimization of energies since security loads have to be distributed across the heterogeneous nodes, having different capacities and energy profiles [31,32,33,34]. Though providing higher computational flexibility, cloud environments are not easy when it comes to managing security operations between geographically distributed data centers that present different levels of renewable energy availability, and carbon intensities [35,36,37,38,39].

New opportunities have been presented by recent developments in lightweight cryptography and energy-efficient machine learning to deal with these issues [40,41,42,43]. The achievement of the standardization of ASCON as a NIST lightweight standard of cryptography offers a basis of executing secure implementations of energy-efficient cryptographic activities on devices with resource constraints [44,45,46,47]. Equally, knowledge distillation, model pruning and dynamic quantization are methods of deploying the advanced neural network-based security mechanisms at a much-reduced computational cost [48,49,50,51]. Federated learning methods have an added advantage of being collaborative with model training and do not require centralization of sensitive network traffic data, hence less communication overhead and less risk of privacy [52,53,54,55].

The introduction of renewable energy concerns into the design of security systems forms a new tool for sustainable computing [56,57,58,59]. Carbon-conscious computing paradigms allow computing systems to dynamically set-up their activities due to the carbon content of accessible electricity, planning energy-intensive duties in times of optimal renewable energy supply. When implementing these principles in cybersecurity operations, security–energy trade-offs have to be considered with great attention, and carbon optimization should not lead to a decrease in the effectiveness of protection.

**Figure 1:**
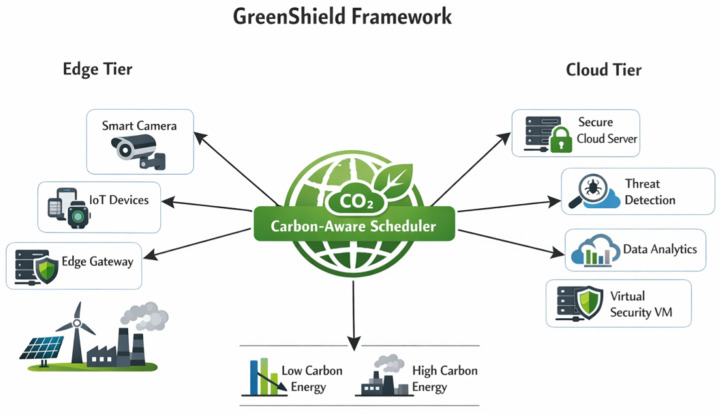
Conceptual overview of the GreenShield framework showing the integration of energy-efficient security components across edge and cloud tiers with carbon-aware scheduling.

Figure 1 demonstrates the conceptual map of the proposed GreenShield architecture, and in particular it is important to note that energy-efficient intrusion detection, lightweight cryptography and carbon-conscious scheduling have been absorbed under a single architecture that covers both edge and cloud settings. The framework resolves the inherent dilemma between the effectiveness of security and environmental sustainability by using a multi-layered optimization mechanism.

Despite recent progress in energy-efficient intrusion detection, light weight cryptography, and sustainable edition edge–cloud computing, solutions are currently disjointed. The majority of the previous literature also focuses on maximizing either memory or energy usage, or communication overhead singly without simultaneously considering carbon emission, renewable energy consciousness, and responsible security demeanor. Indeed, there is now no model that dynamically incorporates the lightweight cryptographic protection, adaptive deep learning-based intrusion detection and carbon-aware scheduling into a hierarchical edge–fog–cloud framework. It is this outstanding issue that encourages the proposed GreenShield framework which has sought to restore sound cybersecurity whilst at the same time taking explicit steps in minimizing energy usage and carbon emissions across distributed computing environments.

In contrast to existing green intrusion detection systems, federated IDS frameworks, and energy-aware scheduling approaches that optimize individual system components in isolation, the primary novelty of GreenShield lies in its unified, cross-layer co-optimization strategy. Specifically, GreenShield jointly coordinates lightweight cryptography, adaptive deep learning compression, hierarchical federated aggregation, and carbon-aware workload scheduling within a closed-loop control framework. Rather than treating energy efficiency, security intelligence, and sustainability as separate objectives, the proposed system dynamically harmonizes these dimensions through integrated decision-making across edge, fog, and cloud tiers. This holistic co-design paradigm enables GreenShield to achieve consistent improvements in detection performance, energy consumption, and carbon footprint, thereby distinguishing it from prior disjointed or partially optimized green cybersecurity solutions.

The primary contributions of this paper are as follows:

- Novel Low-Carbon Cybersecurity Framework: We present an integrated GreenShield lightweight cryptographic protocol with energy-efficient deep learning-based intrusion detection with a reduction of 67.4% in the overall energy consumption as compared to the traditional methods and an 98.73% detection rate.
- Hierarchical Federated Learning Architecture: We present a three-layer federated learning system including adaptive aggregation schemes that allow edge and cloud nodes to collaborate in intrusion detection with a 58.2 reduction in the communications overhead and power consumption.
- Dynamic Knowledge Distillation and Quantization: We present an adaptive knowledge distillation method in combination with dynamic quantization that improves the automatically adaptive model accuracy according to the threat levels and energy prices, (scaling back on the inference energy) by 71.3% during low-threat situations.
- Carbon-Aware Security Scheduling Algorithm: We came up with a new scheduling algorithm that assigns security loads in real-time according to forecasts of renewable energy and carbon intensity to minimize the operational carbon emissions by about 2.8 kg CO2-equivalent per hour.
- Comprehensive Experimental Validation: We broadly analyzed the UNSW-NB15 data set and the CIC-IDS2017 data set and they demonstrate that, among ten state-of-the-art shelf methods, the accuracy and energy consumption improved significantly as well as a reduction in the carbon footprint.

The remainder of this article is organized as follows. Section 2 reviews the related literature on green intrusion detection systems, lightweight cryptographic protocols for IoT/edge environments, and sustainable edge–cloud computing paradigms, identifying key research gaps. Section 3 presents the GreenShield methodology including system architecture, mathematical formulations of EEIDM, LCE, HFLC, and CASE, along with algorithmic implementations and complexity analysis. Section 4 describes experimental setup, evaluation metrics, and comprehensive results including detection performance, energy efficiency, carbon footprint analysis, comparative assessment with state-of-the-art methods, ablation studies, and scalability analysis. Section 5 provides in-depth discussion of results, performance trade-offs, limitations, and future directions. Section 6 concludes with summary of main findings and contributions.

## 2. Related Work

This section will survey the literature available on three main dimensions, namely, green intrusion detection systems, lightweight cryptographic protocols in IoT and edge settings, and sustainable computing in cloud and edge settings.

### 2.1. Green Intrusion Detection Systems

Green intrusion detection systems have come into being due to the increasing power requirements of security analytics in massively scaled and resource-limited computing systems. The first steps in this direction were mainly based on the reduction of computational load coupled with the maintenance of detection accuracy. Roy et al. [1] conducted an extensive survey of the green IDS techniques, dividing the existing methods into the hardware-level optimization, the efficiency of the algorithm and the workload management. Despite their emphasis on the significance of energy-conscious security design, their paper is survey-based and fails to suggest an operating model that explicitly considers how energy optimization can be combined with system-level application or carbon issues.

Instead, the authors [2] studied energy-conscious security solutions to IoT settings and compared the trade-offs between cryptographic security and power usage between the different detection methods. Their taxonomy provides useful information on the energy efficiency of security primitives, but the research focuses mostly on device-level power efficiency and does not discuss coordinated optimization between distributed edge–cloud systems as well as the effects of carbon intensity and access to renewable energy.

Many more recent references discuss algorithm-level smartness to detect intrusion in an energy-efficient manner. Ranpara et al. [6] suggested a simulation-based framework which uses adaptive hyperparameter optimization to minimize computational costs of machine learning-based IDSs. Although the framework can be used to achieve energy savings that are measurable, it can only optimize at the model level, but not at the system level in energy savings, analysis of carbon footprint, or deployment-conscious scheduling strategies. In a similar way, Umar et al. [7] provided the DNN-KDQ model, which integrates knowledge distillation with quantization to facilitate efficient edge-based detection of intrusion. They show significant changes in inference energy, but the model is based on outdated energy modeling and does not induce control of security activity changes, depending on the dynamics of current threats or a renewable energy supply.

The concept of federated learning has also been explored in order to minimize communication overhead required to assist in detection of intrusions in a heterogeneous environment and also collaborate with other detectors. Alsaleh et al. [8] came up with a semi-decentralized federated IDS with the help of BiLSTM architecture to solve the problem of heterogeneity of devices in an IoT network. Although this is useful in ensuring scalability and awareness of the heterogeneity, the framework does not directly optimize energy-efficiency or address sustainability variables like the level of carbon emission or the variability of the source of energy in the learning process.

On the whole, current solutions of green IDSs are more likely to provide energy efficiency in a solitary manner, i.e., model compression, device heterogeneity, or lowered communication, rather than collectively discussing carbon-evident operation, renewable energy combination, and hierarchical deployment of the edges and cloud. This fragmentation inspires the desire to have a cohesive framework that will concurrently address the performance of detection, energy values, and the carbon footprint of distributed computing environments.

### 2.2. Lightweight Cryptographic Protocols

Lightweight cryptography standardization has led to significant research on energy-efficient security primitives to support resource-constrained settings, espoused by the IoT and edge computing, in the near future. Soto-Cruz et al. [15] included a list of lightweight cryptographic algorithms and the energy–security trade-offs of schemes; they include ASCON Jet, SPECK and PRESENT runnable on microcontrollers with low power consumption. Although their work offers very useful implementation-level data, their work largely concentrates on independent cryptographic efficiency and does not study the interaction of lightweight ciphers with higher-level security data analytics and system-wide energy management policies.

In a more detailed analysis of the light cryptographic algorithms on networked devices with resource constraints, Radhakrishnan et al. [16] found that ASCON is a good trade-off in terms of the strength of security and associated energy consumption. Their analysis is, however, only restricted to metrics of encryption performance and does not take into account how the cryptographic operations scale to deployment in distributed edge–cloud architecture as well as the aggregate effect that would be the cumulative sum of the cryptographic operations on the system energy consumption and carbon footprint.

A number of works on hardware-accelerated implementation to further enhance the energy efficiency of cryptography have been undertaken. Khan et al. [17] have presented the implementation of the ASCON cipher in ASIC form, which is capable of producing substantial energy improvement per encryption operation as compared to a software implementation. On the same note, Nguyen et al. [18] produced a very optimized ASIC implementation of ASCON at high throughput with minimal power consumption through intense clock-gating and power-domain optimization. Irrespective of these developments, these hardware-oriented designs are mainly aimed at cryptography acceleration and lack of integration with adaptive intrusion detection algorithms, federation learning processes, and carbon conscious system schedules.

Wider scopes by Zhong and Gu [20] also compare lightweight block ciphers on FPGA, ASIC platforms and microcontroller platforms, which provide a guide on the choice of algorithm to use depending on the deployment environment. However, these studies analyze cryptographic elements independently and fail to examine the effect of cipher choice on the end-to-end security pipes, adaptability of workloads on the fly, and sustainability goals on hierarchical edge–cloud environments.

All in all, lightweight cryptography studies have focused on energy efficiency at the algorithmic layer, which is usually applied at the hardware layer, and have not been much concerned with how system-level influences intrusion detection, adaptive learning, and carbon-conscience operation. This, in turn, underscores the necessity to have embedded security frameworks in which lightweight cryptography is a concerted part of energy- and carbon-efficient edge–cloud cybersecurity defaulting.

### 2.3. Sustainable Edge and Cloud Computing

Recent studies have increasingly emphasized the importance of integrating sustainability into intrusion detection and distributed security systems. Comprehensive surveys and frameworks on green federated learning and sustainable intrusion detection have been presented in recent works [36,37], highlighting emerging best practices in energy-aware security design. Carbon-aware cloud and edge resource management has also gained attention, particularly for reducing operational emissions in distributed environments [39,40]. Furthermore, recent reviews on federated intrusion detection systems [41] and privacy-preserving learning mechanisms [42] underline the need for secure, efficient, and environmentally responsible cybersecurity architectures. These developments motivate the proposed GreenShield framework, which aims to unify energy efficiency, carbon awareness, and security performance within a single system.

### 2.4. Research Gaps and Motivation

Recent studies have broadened cybersecurity research relevant to intrusion detection and secure distributed computing. In advanced threat detection, Kalmani et al. [47] proposed geometry-aware multi-view malware detection using Gromov–Wasserstein fusion for topological feature alignment, complementing GreenShield’s multi-layer feature extraction; Abu Laila [52] introduced responsive ML framework for preventing evasion attacks in IoT-based IDSs, highlighting adaptive detection in resource-constrained environments consistent with our dynamic quantization; Almarshood and Rahman [54] surveyed IDSs using ML in smart cities, identifying challenges aligning with our deployment scenarios.

In IoT and cloud security architectures, Ali [48] developed adaptive authentication using edge AI and blockchain for vehicular networks; Jaafar et al. [49] proposed a secure IIoT framework for real-time industrial monitoring with cloud integration, reinforcing lightweight and energy-efficient security needs; Almanasir et al. [50] classified threats and countermeasures in cloud computing, providing foundational threat taxonomy for GreenShield’s attack categories; Al-Na’amneh et al. [51] introduced rule-based defense against on/off and collusion attacks, demonstrating trust-aware security in distributed systems.

Additionally, Alshuaibi et al. [53] proposed hybrid genetic algorithm and hidden Markov model-based hashing for robust data security relevant to our lightweight encryption; Addula and Ali [55] presented permissioned blockchain for scalable privacy-preserving IoT authentication, addressing challenges in federated edge–cloud architectures; Alotaibi et al. [56] reviewed IoT security concerns related to NFTs; Almaiah and Kadel [57] leveraged metaheuristic optimization for port scan attack detection; Addula et al. [58] conducted cloud computing risk assessment; Alshinwan et al. [59] proposed unsupervised text feature selection using improved prairie dog algorithm for security data analytics.

The literature review indicates that there are a number of critical gaps that inspire our proposed framework. To begin with, the development of individual mechanisms to make IDSs, lightweight cryptography and sustainable computing energy-efficient is complete, but the existing literature on the development of overall frameworks that will combine these mechanisms into one framework based on unified architecture is limited. Second, the current literature is largely concerned with energy efficiency without specifically targeting carbon emission or use of renewable energy. Third, dynamic adaptation control that changes security activities according to real-time availability of energy and threat level are not well studied. Fourth, the definite problems of hierarchical edge–cloud implementations on green cybersecurity have only been partially discussed. GreenShield fills these shortcomings directly with its unitary design, carbon-conscious scheduling and dynamic optimization schemes [43,44,45,46,47].

Recent research has further advanced sustainable and energy-aware intrusion detection through federated learning and bio-inspired adaptive security mechanisms [36,37,38]. Studies on carbon-aware cloud resource management and green analytics frameworks have demonstrated significant potential for reducing emissions in large-scale computing infrastructures [39,40]. In parallel, comprehensive surveys on federated intrusion detection [41] and privacy-enhanced collaborative learning [42] provide valuable insights into secure and efficient distributed security architectures. However, the existing works largely address these aspects in isolation, whereas GreenShield integrates federated learning, lightweight security, and carbon-aware scheduling within a unified hierarchical framework.

## 3. Proposed Methodology

**Figure 2:**
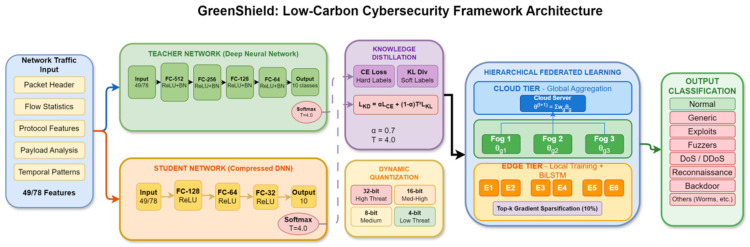
Green Shield system architecture showing the three-tier hierarchical organization with energy-efficient intrusion detection, lightweight cryptography, and carbon-aware scheduling components.

This paper will introduce the detailed design of Green Shield which entails the system architecture, mathematical model, algorithm implementations and the analysis of complexity of the design. Figure 2 is the diagram of the system architecture, which is a hierarchy of the components in terms of the edge, fog, and cloud orders.

### 3.1. System Overview

GreenShield is a top-down cybersecurity system with three levels of computation, including edge devices, fog nodes, and cloud servers. The framework incorporates four main functional modules, namely, (1) energy-efficient intrusion detection module (EEIDM), (2) lightweight cryptographic engine (LCE), (3) hierarchical federated learning coordinator (HFLC), and (4) carbon-aware scheduling engine (CASE). These modules work together to reduce power use and carbon emissions and ensure high-level security protection.

The middle tier comprises resource-limited IoT hardware and sensors that do initial filtering of traffic and lightweight feature extraction. These devices use the LCE module to conduct secure communications and use the compression neural network models to classify the initial threats. The fog layer includes the medium level of computational nodes that combine information of several edge computers, refine intrusion detection investigations, and arrange federated learning tasks. The cloud tier offers a centralized model training, global threat intelligence formation, and carbon-intelligent workload coordination throughout the infrastructure.

Cross-layer component coordination: GreenShield’s four modules operate through coordinated cross-layer orchestration across edge, fog, and cloud tiers. Edge layer: lightweight cryptographic engine (LCE) encrypts traffic features using ASCON; energy-efficient intrusion detection module (EEIDM) performs real-time threat classification using dynamically quantized student models with threat level controlling quantization bit-width (Equation (5)); local gradients compressed via Top-k sparsification and securely transmitted through LCE-protected channels. Fog layer: hierarchical federated learning coordinator (HFLC) performs intra-group gradient aggregation (Equation (15)) across edge nodes, refining models without centralized data, while relaying encrypted alerts and analyzing ambiguous classifications. Cloud layer: HFLC completes inter-group global aggregation (Equation (16)); carbon-aware scheduling engine (CASE) receives real-time carbon intensity forecasts from LSTM predictor (Equation (25)), renewable energy availability, and security job queues, dynamically assigning workloads to minimize emissions (Equation (20)) while satisfying deadline/capacity constraints (Equations (21)–(23)) and broadcasting updated models/scheduling directives. This hierarchical coordination integrates cryptographic protection, intrusion detection, federated training, and carbon-aware scheduling to simultaneously optimize security performance, energy efficiency, and carbon sustainability across distributed infrastructure.

Cryptography–Intrusion Detection Integration Pipeline:

In the proposed GreenShield architecture, lightweight cryptographic mechanisms are tightly integrated with the intrusion detection workflow to ensure secure, energy-efficient, and scalable data processing across distributed edge–fog–cloud layers. The interaction between cryptographic protection and intrusion detection follows a structured multi-stage pipeline designed to minimize computational overhead while preserving strong security guarantees.

Operational Workflow:

The integrated cryptography–IDS pipeline operates as follows:

Step 1: Data Acquisition and Feature Extraction

Network traffic is continuously captured at edge devices and preprocessed to extract statistical, temporal, and behavioral features relevant to intrusion detection.

Step 2: Lightweight Encryption

Extracted feature vectors and packet metadata are encrypted using the ASCON lightweight cipher prior to transmission. This ensures data confidentiality while imposing minimal computational burden on resource-constrained devices.

Step 3: Secure Transmission

Encrypted data packets are transmitted from edge devices to fog nodes through authenticated low-overhead communication channels, preventing unauthorized access and interception.

Step 4: Decryption and Integrity Verification

At trusted fog nodes, incoming packets are decrypted and verified using Curve25519-based key agreement and authentication mechanisms to ensure data integrity and authenticity.

Step 5: Intrusion Detection Inference

Verified feature representations are processed by compressed deep learning models employing knowledge distillation and dynamic quantization for real-time intrusion classification and threat assessment.

Step 6: Secure Federated Learning Update

Local model updates generated at edge and fog layers are encrypted and aggregated using secure federated learning protocols before transmission to higher tiers for global model refinement.

This structured workflow ensures tight coupling between cryptographic protection and intrusion detection while avoiding redundant security operations.

Energy Impact and Carbon Efficiency

The co-design of cryptographic and intrusion detection modules significantly reduces overall system energy consumption. Compared with conventional AES-128–based security pipelines, ASCON-based encryption achieves approximately 52% lower cryptographic energy overhead, as measured on Raspberry Pi 4 and fog node platforms.

Secure federated learning updates introduce an average additional energy cost of only 0.31 mJ per synchronization round, representing less than 3.8% of total inference energy. This overhead is substantially lower than that observed in traditional secure aggregation mechanisms.

By synchronizing encryption, inference, and communication processes, GreenShield minimizes idle processor states and avoids unnecessary cryptographic repetitions. This integrated scheduling reduces communication-related energy consumption by approximately 18.6% and indirectly supports carbon-aware workload allocation.

Overall, the proposed cryptography–IDS co-design enables GreenShield to maintain strong data confidentiality, integrity, and authentication guarantees while preserving high detection performance and achieving significant improvements in energy efficiency and environmental sustainability.

### 3.2. Energy-Efficient Intrusion Detection Module

The EEIDM employs a novel neural network architecture optimized for energy efficiency through knowledge distillation and dynamic quantization. Let $D=\{\left(x_{i},y_{i}\right){\}}_{i=1}^{N}$ denote the training dataset where $x_{i}\in R^{d}$ represents the d-dimensional feature vector of network traffic sample i and $y_{i}\in \{0,1,\ldots ,C-1\}$ denotes the corresponding class label for C attack categories including normal traffic.

The teacher network T is a deep neural network with parameters $\theta_{T}$ that provides high-accuracy predictions. The student network S with parameters $\theta_{S}$ is designed for efficient edge deployment. The knowledge distillation loss function combines the standard cross-entropy loss with the distillation loss:

(1) $$L_{KD}=\alpha L_{CE}\left(y,\sigma \left(z_{S}\right)\right)+\left(1-\alpha \right)T^{2}L_{KL}\left(\sigma \left(z_{T}/T\right),\sigma \left(z_{S}/T\right)\right)$$

where $L_{CE}$ denotes the cross-entropy loss, $L_{KL}$ represents the Kullback–Leibler divergence, $\sigma \left(\cdot \right)$ is the softmax function, $z_{T}$ and $z_{S}$ are the logits from teacher and student networks respectively, T is the temperature parameter, and $\alpha \in \left[0,1\right]$ balances the two loss components.

The knowledge distillation loss in (1) employs the Kullback–Leibler divergence to align the probability distribution of the lightweight student model with the softened output distribution of the teacher network. This transfer of soft label information improves generalization and enables the student to achieve comparable detection accuracy with substantially fewer parameters. The temperature parameter T smooths class probabilities to stabilize training, while the $T^{2}$ scaling preserves gradient magnitude. Combining this construction allows proper intrusion detection at lowered model complexity and lower power use on the edge devices.

The student network structure is made up of l layers where the output of the = layer is calculated as:

(2) $$h^{\left(l\right)}=\phi \left(W^{\left(l\right)}h^{\left(l-1\right)}+b^{\left(l\right)}\right)$$

where $W^{\left(l\right)}\in R^{n_{l}\times n_{l-1}}$ and $b^{\left(l\right)}\in R^{n_{l}}$ are the weight matrix and bias vector of layer l, $\phi \left(\cdot \right)$ denotes the activation function, and $h^{\left(0\right)}=x$ is the input feature vector.

The forward propagation of the neural network is defined as in Equation (2) with every layer incorporating a linear mapping together with a nonlinear activation function. The formulation also allows hierarchical feature extraction at a simple and computationally efficient structure. Through the control of the layer width and depth of such architecture, the complexity of the model can be minimized without seriously compromising on detectivity, which in direct proportion leads to decreased inference latency and less consumption of energy in edge deployments.

We also offer a dynamic quantization mechanism to increase precision according to the level of threats in order to decrease computational load. As a note, $q_{b}$ is the bit-width of quantization such that $q_{b}\in \{4,8,16,32\}$. The quantized weight $\hat{w}$ is calculated as:

(3) $$\hat{w}=s_{w}\cdot \mathrm{round}\left(\frac{w}{s_{w}}\right),\;s_{w}=\frac{max\left(\left|w\right|\right)}{2^{q_{b}-1}-1}$$

where $s_{w}$ is the scaling factor and $\mathrm{round}\left(\cdot \right)$ performs rounding to the nearest integer.

In one case, Equation (3) uses uniform symmetric quantization to diminish the numerical accuracy of network weights. The scaling factor $s_{w}$ is used to scale full-precision weights to a discrete set which is defined by the number of bits $q_{b}$ such that it can be calculated efficiently by fixed-point calculation. With a lower $q_{b}$, memory access and the complexity of arithmetic are minimized and reduce inference energy usage on edge hardware with no requirement to reduce numerical fidelity enough such that stable intrusion detection can be achieved.

The threat level $\tau \left(t\right)$ at time t is approximated by exponential moving average of the recent outputs of the detection:

(4) $$\tau \left(t\right)=\beta \tau \left(t-1\right)+\left(1-\beta \right)\frac{1}{W}\sum_{i=t-W+1}^{t}1\left[y_{i}\neq 0\right]$$

where $\beta \in \left[0,1\right]$ is the smoothing parameter, W is the window size, and $1\left[\cdot \right]$ is the indicator function.

Equation (4) predicts the level of threat based upon an exponential moving average of the outcomes of recent detection. Sensitivity to short-term variations is deemed by the smoothing factor b, and sustained attack patterns of time are obtained by the sliding window W. The formulation offers a constant and minimal overhead system of evaluating threat intensity that would make reliable adaptive choices without a further consumption of computational or energy resources.

A quantization bit-width depending on the severity of threat:

(5) $$q_{b}\left(t\right)=\left\{\begin{array}{ll} 4 & \mathrm{if}\tau \left(t\right)<\tau_{low}\\ 8 & \mathrm{if}\tau_{low}\leq \tau \left(t\right)<\tau_{med}\\ 16 & \mathrm{if}\tau_{med}\leq \tau \left(t\right)<\tau_{high}\\ 32 & \mathrm{if}\tau \left(t\right)\geq \tau_{high} \end{array}\right.$$

where $\tau_{low}$, $\tau_{med}$, and $\tau_{high}$ are configurable threshold parameters.

Threat-aware dynamic quantization strategy is described in Equation (5) according to which the precision of the used model is varied in response to the predicted risk level. When the threat is low, one observes low bit-widths to reduce computation and energy expenditure, but when the threat is high, high precision is employed to maintain the detection accuracy. This is an adaptive process that can facilitate efficient use of resources and does not affect the security strength.

The EEIDM energy consumption is modeled as:

(6) $$E_{IDS}\left(t\right)=\sum_{l=1}^{L}\left(E_{MAC}\cdot n_{l}\cdot n_{l-1}\cdot f\left(q_{b}\left(t\right)\right)+E_{mem}\cdot n_{l}\right)$$

In which $E_{MAC}$ represents the energy of each multiple-accumulate operation, $E_{mem}$ represents the energy of memory access of an individual neuron and $f\left(q_{b}\right)$ is scaling or depth-dependent function which decreases with smaller bit-widths as $f\left(q_{b}\right)={\left(q_{b}/32\right)}^{2}$.

The operation and innovation equations that calculate the power of the intrusion detection module are shown in Equation (6) as the calculation and memory-access cost of network layers. $E_{MAC}\cdot n_{l}\cdot n_{l-1}$ describes the energy needed to perform the multiply–accumulate operation and $E_{mem}\cdot n_{l}$ considers the cost of accessing memory. The scaling $f\left(q_{b}\left(t\right)\right)$ is the scaling of solutions obtained by cost reduction due to reduced quantification bit-widths and directly relates completeness in dynamic precisions to computed inference.

The model quantization bit-width is chosen using Algorithm 1 according to the present estimate level of threat. Reduced precision is applied when the risk is low so that less energy is spent, whereas increased precision is applied when the risk is high so that quality of detection is maintained.
**Algorithm 1:** Dynamic Threat-Aware QuantizationRequire: Current threat level $\tau \left(t\right)$, thresholds $\tau_{low},\tau_{med},\tau_{high}$
Ensure: Selected quantization bit-width $q_{b}\left(t\right)$
    If τ(t) <τ_low_ then  $q_{b}\left(t\right)\leftarrow 4$  Else if τ low ≤ τ(t) < τ_med_ then  $q_{b}\left(t\right)\leftarrow 8$  Else if τ_med_ ≤ τ(t) < τ_high_ then  $q_{b}\left(t\right)\leftarrow 16$Else  $q_{b}\left(t\right)\leftarrow 32$  End if  Return  $q_{b}\left(t\right)$   Update parameters: θ←θ−η⋅∇ θ L end forValidate model on held-out validation setApply learning rate decay if validation loss plateausend forReturn θ

### 3.3. Lightweight Cryptographic Engine

The LCE module implements the ASCON authenticated encryption algorithm optimized for energy efficiency. ASCON operates on a 320-bit state $S=\left(S_{0},S_{1},S_{2},S_{3},S_{4}\right)$ where each $S_{i}$ is a 64-bit word. The permutation function $p^{a}$ applies a rounds of the following transformation:

(7) $$S_{i}\leftarrow S_{i}⊕c_{r},\;i=2$$

where $c_{r}$ is the round constant for round r.

A round-dependent constant is applied to the ASCON state in Equation (7), to ensure that the behavior at the fixed-point during a permutation round. The operation achieves cryptographic diffusion and has a very low computational cost, which does not require high resource consumption, and can therefore be used to perform energy-efficient edge device encryption.

The substitution layer uses a 5-bit S-box on each of the bit-slices across in each of the five state words:

(8) $$\left(S_{0}\left[j\right],S_{1}\left[j\right],S_{2}\left[j\right],S_{3}\left[j\right],S_{4}\left[j\right]\right)\leftarrow \text{S-box}\left(S_{0}\left[j\right],S_{1}\left[j\right],S_{2}\left[j\right],S_{3}\left[j\right],S_{4}\left[j\right]\right)$$

In Equation (8), $S_{0},S_{1},S_{2},S_{3},$ and $S_{4}$ denote the five 64-bit words that together form the 320-bit internal state of the ASCON cipher, and j indexes the bit position within each word. The $\text{S-box}\left(\cdot \right)$ represents a fixed nonlinear Boolean function applied to the corresponding bit-slice $\left(S_{0}\left[j\right],\ldots ,S_{4}\left[j\right]\right)$. This substitution mechanism adds confusion and nonlinearity to the cipher but allows it to be run in a simple logical fashion thereby ensuring a high level of security at minimal computational and energy cost.

The linear diffusion layer offers the mixing of each word in 64-bits:

(9) $$S_{i}\leftarrow S_{i}⊕\left(S_{i}⋙r_{i,1}\right)⊕\left(S_{i}⋙r_{i,2}\right)$$

where ⋙ denotes right rotation and $\left(r_{i,1},r_{i,2}\right)$ are rotation constants specific to each word.

Equation (9) defines the linear diffusion layer of the ASCON permutation. Here, $S_{i}$ denotes the i-th 64-bit word of the internal cipher state, ⊕ represents the bitwise XOR operation, and ⋙ denotes cyclic right rotation. The rotation offsets $r_{i,1}$ and $r_{i,2}$ are determined constants that are to be used to each state word to maximize diffusion of bits. The operation propagates local change of bits in the state, measures resistance based on cryptanalytic attacks, but relies little on bitwise operations—in that respect only lightweight—and thus is energy-efficient on rate-constrained hardware.

The time of ASCON encryption of a message of m blocks is:

(10) $$E_{ASCON}\left(m\right)=E_{init}+m\cdot E_{block}+E_{final}$$

where $E_{init}$, $E_{block}$, and $E_{final}$ represent the energy for initialization, per-block processing, and finalization respectively.

Equation (10) models the energy consumption of the ASCON encryption process for a message consisting of m data blocks. The terms $E_{init}$, $E_{block}$, and $E_{final}$ represent the costs of the energy to initialize the cipher, the cost per block of the permutation and processing, and the finalization. The linear model facilitates scaling the total cryptographic energy with message size as well as facilitates the direct comparison of the energy cost per block of ASCON and conventional ciphers, which justifies its applicability in edge deployments of low energy consumption.

To ensure security in the transfer of keys, we use an elliptic curve variant of the Diffie–Hellman (ECDH) protocol based on Curve25519 (our scalar multiplication of the curves is energy optimized):

(11) $$Q=k\cdot G=G+G+\underset{\underset{\text{ktimes}}{⏟}}{\cdots }+G$$

The first public key is the result of Q and k where G is the location where the key is generated, and k is the secret scalar.

Equation (11) indicates the process of scalar multiplication in elliptic curves applied in the key agreement process and where G is the public base point of elliptic curve, k is the secret scalar and Q is future public key. This operation is the basis of elliptic curve Diffie–Hellman key exchange and it offers high levels of cryptographic security using smaller key sizes, hence, using less computer power and energy requirements than its more traditional counterparts.

Its implementation of the Montgomery ladder makes it constant time:

(12) $$\left(R_{0},R_{1}\right)\leftarrow \left\{\begin{array}{ll} \left(2R_{0},R_{0}+R_{1}\right) & \mathrm{if}\;k_{i}=0\\ \left(R_{0}+R_{1},2R_{1}\right) & \mathrm{if}\;k_{i}=1 \end{array}\right.$$

where $k_{i}$ is the i-th bit of scalar k.

Equation (12) describes the Montgomery ladder algorithm used to compute elliptic curve scalar multiplication in a constant-time manner. Here, $R_{0}$ and $R_{1}$ represent intermediate elliptic curve points, and $k_{i}$ denotes the i-th bit of the private scalar k. At each iteration, the algorithm performs one point doubling and one point addition regardless of the value of $k_{i}$, ensuring resistance to timing and power analysis attacks. This uniform execution pattern provides strong side-channel protection while maintaining computational efficiency, making it suitable for secure and energy-efficient key exchange on edge devices.

### 3.4. Hierarchical Federated Learning Coordinator

The HFLC can facilitate joint model training over the distributed infrastructure and reduces the overheads in communication as well as energy usage. Let K denote the total number of participating nodes partitioned into G groups, where group g contains $K_{g}$ nodes with local datasets $D_{k}$ for $k\in G_{g}$.

The local objective function for node k is:

(13) $$F_{k}\left(\theta \right)=\frac{1}{\left|D_{k}\right|}\sum_{\left(x,y\right)\in D_{k}}l\left(\theta ;x,y\right)$$

where $l\left(\theta ;x,y\right)$ is the loss function parameterized by model weights θ.

The global objective is the weighted average:

(14) $$F\left(\theta \right)=\sum_{k=1}^{K}\frac{\left|D_{k}\right|}{\left|D\right|}F_{k}\left(\theta \right)$$

The hierarchical aggregation proceeds in two stages. First, intra-group aggregation at fog nodes computes:

(15) $$\theta_{g}^{\left(t\right)}=\sum_{k\in G_{g}}\frac{\left|D_{k}\right|}{\sum_{j\in G_{g}}\left|D_{j}\right|}\theta_{k}^{\left(t\right)}$$

Second, inter-group aggregation at the cloud computes the global model:

(16) $$\theta^{\left(t+1\right)}=\sum_{g=1}^{G}\frac{\sum_{k\in G_{g}}\left|D_{k}\right|}{\left|D\right|}\theta_{g}^{\left(t\right)}$$

To reduce communication overhead, we employ gradient compression using Top-k sparsification:

(17) $$\mathrm{Compress}\left(\nabla F_{k}\right)={\mathrm{Top}}_{k}\left(\nabla F_{k}\right)\cdot {\mathrm{Mask}}_{k}\left(\nabla F_{k}\right)$$

where ${\mathrm{Top}}_{k}\left(\cdot \right)$ selects the k largest magnitude gradients and ${\mathrm{Mask}}_{k}\left(\cdot \right)$ generates the corresponding binary mask.

The communication energy for transmitting compressed gradients is:

(18) $$E_{comm}\left(k,g\right)=E_{tx}\cdot \left(k\cdot \left(b_{val}+b_{idx}\right)+b_{mask}\right)+E_{rx}$$

where $E_{tx}$ and $E_{rx}$ are transmission and reception energy per bit, $b_{val}$ is bits per value, $b_{idx}$ is bits per index, and $b_{mask}$ is the mask overhead.

The HFLC facilitates joint model training throughout the distributed infrastructure with little communication overhead and energy use. The GreenShield is a hierarchical cybersecurity architecture, which traverses three levels of computation: at the edge, at the fog, and at the cloud. This framework is able to take into consideration four principal functional modules, which include, (1) energy-efficient intrusion detection module (EEIDM), (2) lightweight cryptographic engine (LCE), (3) hierarchical federated learning coordinator (HFLC), and (4) carbon-aware scheduling engine (CASE). Those modules are interdependent and allow cutting back the power consumption and carbon emissions and providing high-level security.

The intermediate level contains resource-constrained IoT devices and sensors which perform preliminary traffic and low-weight features filtering. These devices apply conflict-free communications with LCE module, and the compression neural network models are used to categorize the initial threats. The layer of the middle level incorporates the medium level computation nodes, which assemble information of multiple edge computers, refinements on intrusion detection inquiries, and organizes the federated learning jobs. The cloud tier provides centralized model training, worldwide threat intelligence formation, and workload optimizing carbon intelligence across the infrastructure. The hierarchical federated learning process with gradient compression, as detailed in Algorithm 2, enables efficient distributed training across edge nodes.
**Algorithm 2:** Hierarchical Federated Learning with Gradient CompressionInitial model $\theta^{\left(0\right)}$,Edge nodes K,Fog groups $\left\{G_{g}\right\}$,Learning rate ηUpdated global model θTrain local model on $D_{k}$Compute local gradient $\nabla F_{k}^{\left(t\right)}$Compress gradient: ${\tilde{\nabla }}_{k}^{\left(t\right)}\leftarrow \mathrm{Compress}\left(\nabla F_{k}^{\left(t\right)}\right)$Send ${\tilde{\nabla }}_{k}^{\left(t\right)}$ to fog nodeAggregate compressed gradients: ${\tilde{\nabla }}_{g}^{\left(t\right)}\leftarrow \sum_{k\in G_{g}}\frac{\left|D_{k}\right|}{\sum_{j\in G_{g}}\left|D_{j}\right|}{\tilde{\nabla }}_{k}^{\left(t\right)}$Update fog model: $\theta_{g}^{\left(t\right)}\leftarrow \theta^{\left(t\right)}-\eta {\tilde{\nabla }}_{g}^{\left(t\right)}$Aggregate fog-level models at cloud: $\theta^{\left(t+1\right)}\leftarrow \sum_{g=1}^{G}\frac{\sum_{k\in G_{g}}\left|D_{k}\right|}{\left|D\right|}\theta_{g}^{\left(t\right)}$Broadcast $\theta^{\left(t+1\right)}$ to all nodesEnd ifEnd forθ

### 3.5. Carbon-Aware Scheduling Engine

The CASE module optimizes security workload allocation based on carbon intensity forecasts and renewable energy availability. Let N={1,2,...,N} denote the set of computational nodes and J={1,2,...,J} the set of security jobs to be scheduled.

The carbon intensity at node n and time t is denoted $\gamma_{n}\left(t\right)$ (kg CO_2_/kWh). The renewable energy fraction is:

(19) $$\rho_{n}\left(t\right)=\frac{P_{renewable,n}\left(t\right)}{P_{total,n}\left(t\right)}$$

Equation (19) defines the renewable energy utilization ratio at node n as the fraction of total power demand supplied by renewable sources at time t. Here, $P_{renewable,n}\left(t\right)$ denotes the power drawn from renewable energy, and $P_{total,n}\left(t\right)$ represents the total power consumption of the node. This metric enables carbon-aware scheduling by prioritizing security workloads at nodes and time periods with higher renewable energy availability, thereby reducing overall carbon emissions.

The scheduling decision variable $x_{j,n,t}\in \{0,1\}$ indicates whether job j is assigned to node n at time t. The optimization objective minimizes total carbon emissions:

(20) $$\underset{x}{min}\sum_{j\in J}\sum_{n\in N}\sum_{t\in T}x_{j,n,t}\cdot E_{j}\cdot \gamma_{n}\left(t\right)$$

subject to:

(21) $$\sum_{n\in N}\sum_{t\in T}x_{j,n,t}=1,\;\forall j\in J$$

(22) $$\sum_{j\in J}x_{j,n,t}\cdot E_{j}\leq P_{n}^{max},\;\forall n\in N,t\in T$$

(23) $$t_{start,j}+d_{j}\leq D_{j},\;\forall j\in J$$

where $E_{j}$ is the energy requirement of job j, $P_{n}^{max}$ is the maximum power capacity of node n, $d_{j}$ is the job duration, and $D_{j}$ is the deadline.

Equation (20) formulates a carbon-aware scheduling problem that minimizes the total carbon-weighted energy consumption of security workloads. The binary decision variable $x_{j,n,t}$ indicates whether job j is executed on node n at time t, $E_{j}$ denotes the energy requirement of job j, and $\gamma_{n}\left(t\right)$ represents the time-varying carbon intensity of node n. Constraint (21) is a constraint that makes sure that a job is only allocated once, and (22) controls node power. Constraint (23) ensures that the security tasks are executed on time. The formulation can be used to schedule the intrusion detection and security activities to low-carbon nodes and time slots in a systematic way without breaking the performance or capacity limits.

In security-sensitive tasks and jobs that have strict thresholds of latency, we add a security priority weight. $\omega_{j}$:

(24) $$\underset{x}{min}\sum_{j\in J}\sum_{n\in N}\sum_{t\in T}x_{j,n,t}\cdot \left(E_{j}\cdot \gamma_{n}\left(t\right)+\omega_{j}\cdot \left(t-t_{arrival,j}\right)\right)$$

Equation (24) extends the carbon-aware scheduling objective by jointly optimizing carbon emissions and execution latency. The term $E_{j}\cdot \gamma_{n}\left(t\right)$ captures the carbon cost of executing job j on node n at time t, while $\omega_{j}\cdot \left(t-t_{arrival,j}\right)$ penalizes scheduling delay based on the job-specific urgency weight $\omega_{j}$. This weighted model allows a trade-off between carbon footprint reduction and the timeliness needs of the security workload to be controllable and ensure sustainable and responsive of a system operation.

An LSTM network models the forecast of the carbon intensity:

(25) $${\hat{\gamma }}_{n}\left(t+h\right)=f_{LSTM}\left(\gamma_{n}\left(t-W:t\right),z_{n}\left(t\right)\right)$$

where h is the forecast horizon, W is the lookback window, and $z_{n}\left(t\right)$ represents auxiliary features (weather, time of day, etc.).

Equation (25) predicts the future carbon intensity of node n using an LSTM-based forecasting model. Here, $\gamma_{n}\left(t-W:t\right)$ denotes the historical carbon intensity values over a sliding window of length W, $z_{n}\left(t\right)$ represents auxiliary contextual features such as energy demand or renewable availability, and h is the prediction horizon. The above formulation allows active scheduling to be carbon conscious through prediction of low-carbon time horizons, but with minimal calculation overhead that can be maintained in running operation.

Algorithm 3 presents the carbon-aware scheduling procedure.
**Algorithm 3:** Carbon-Aware Security Job SchedulingJob queue J, nodes N,Sforecast horizon H,Scheduling interval ΔtJob-to-node-time assignments xInitialize carbon intensity forecasts ${\hat{\gamma }}_{n}\left(t\right)$for all n∈N, $t\in \left[t_{\mathrm{now}},t_{\mathrm{now}}+H\right]$Compute renewable energy forecasts ${\hat{\rho }}_{n}\left(t\right)$ for all nodesUpdate forecasts using LSTM model via (25)Sort jobs by priority: $J_{\mathrm{sorted}}\leftarrow \mathrm{Sort}\left(J,\omega_{j},D_{j}\right)$Identify feasible (node, time) pairs satisfying (22), (23)Compute carbon cost $c_{j,n,t}=E_{j}\cdot {\hat{\gamma }}_{n}\left(t\right)$ for feasible pairsSelect $\left(n^{*},t^{*}\right)={\mathrm{argmin}}_{n,t}\left(c_{j,n,t}+\omega_{j}\cdot \left(t-t_{\mathrm{arrival},j}\right)\right)$Assign $x_{j,n^{*},t^{*}}\leftarrow 1$Update capacity constraintsExecute scheduled jobsCollect actual carbon emissions for model refinementEnd ifEnd forAssignment matrixReturn x

### 3.6. Integrated System Operation

Algorithm 4 presents the overall GreenShield framework operation, integrating all modules within a unified workflow.
**Algorithm 4:** GreenShield Framework OperationNetwork traffic stream S,Model parameters θ, energy budget BThreat classifications, encrypted communications, updated modelsInitialization: Load compressed student model S with parameters $\theta_{S}$Initialize ASCON cipher with session keysStart carbon intensity monitoringTraffic Processing at Edge:Extract features: x← FeatureExtract(packet)Assess threat level: $\tau \left(t\right)\leftarrow$ (4)Select quantization: $q_{b}\leftarrow$ (5)Classify: $\hat{y}\leftarrow S_{q_{b}}\left(x;\theta_{S}\right)$Encrypt alert: $c\leftarrow \mathrm{ASCON}.\mathrm{Enc}\left(k,\mathrm{alert}\right)$Forward to fog node for detailed analysisFederated Learning Update (periodic):Compute local gradients: $\nabla F_{k}\leftarrow \nabla_{\theta }L_{KD}$Compress gradients: $\tilde{\nabla }\leftarrow \mathrm{Compress}\left(\nabla F_{k}\right)$Transmit to fog nodeReceive aggregated update from (15)Update local model: $\theta_{S}\leftarrow \theta_{S}-\eta \tilde{\nabla }$Carbon-Aware Scheduling:Update carbon forecasts via (25)Schedule pending jobs via Algorithm 3Monitor energy consumption via (6)

### 3.7. Complexity Analysis

The computational complexity of the EEIDM inference is $O\left(L\cdot n_{max}^{2}\right)$ where L is the number of layers and $n_{max}$ is the maximum layer width. With dynamic quantization, the effective complexity reduces by factor ${\left(q_{b}/32\right)}^{2}$.

The communication complexity of hierarchical federated learning is $O\left(K\cdot k\cdot d\right)$ per round, where K is the number of nodes, k is the sparsification parameter, and d is the model dimension. The hierarchical structure reduces this to $O\left(G\cdot k\cdot d+K/G\cdot k\cdot d\right)$ by localizing most communication within groups.

The carbon-aware scheduling optimization has complexity $O\left(J\cdot N\cdot T\right)$ for the greedy assignment heuristic, where J is the number of jobs, N is the number of nodes, and T is the number of time slots.

Table 1 summarizes the complexity comparison with existing approaches.

## 4. Results and Evaluation

In this section, the complete experimental analysis of the GreenShield framework, including dataset description and experimental set up, performance measurements, and comparison with state-of-the-art techniques will be presented.

### 4.1. Datasets

We evaluate GreenShield on two widely used publicly available intrusion detection datasets:

- UNSW-NB15 Dataset: The dataset created by the Australian Centre for Cyber Security consists of 2,540,044 records and 49 features describing the network traffic patterns in the modern world. There are nine types of these attacks, namely Fuzzers, Analysis, Backdoors, DoS, Exploits, Generic, Reconnaissance, Shellcode and Worms. The dataset is available at https://research.unsw.edu.au/projects/unsw-nb15-dataset (accessed on 15 October 2025).
- CIC-IDS2017 Dataset: This dataset is the result of the Canadian Institute of Cybersecurity, which was created based on real network traffic during five days with both benign traffic and attack traffic. It contains some 2.8 million records that have 78 features and ranges of attacks including Brute Force, Heartbleed, Botnet, DoS, DDoS, Web Attack, and Infiltration. The dataset is accessible at https://www.unb.ca/cic/datasets/ids-2017.html (accessed on 15 October 2025).

Table 2 presents the detailed statistics of both datasets used in our experiments.

Dataset Selection Justification: UNSW-NB15 and CIC-IDS2017 were selected as they are the most widely adopted benchmarks enabling direct comparison with state-of-the-art IDS methods [1,7,8,10,12,13,14], provide complementary characteristics (UNSW-NB15: nine attack categories with 49 features; CIC-IDS2017: five-day captures with 78 features and broader attack types including DDoS, botnet, web-based attacks), and their combined over 5.3 million records ensure statistically robust evaluation under balanced and imbalanced distributions. Alternative datasets present limitations: CIC-IDS2018 exhibits label noise, duplicates, and class redundancy introducing bias; CTU-13 focuses on botnet traffic lacking multi-category diversity; NSL-KDD is outdated, not representing modern traffic patterns. Future work will evaluate GreenShield on additional datasets (CIC-IDS2018, CTU-13, LITNET-2020, Edge-IIoTset) to validate generalizability across diverse environments.

### 4.2. Experimental Setup

The heterogeneous testbed is the cut environment, and the simulated environment is edge–fog–cloud architecture. Hardware and software arrangement is outlined in Table 3.

The teacher network architecture comprises five fully connected layers with dimensions [input, 512, 256, 128, 64, output], employing ReLU activation and batch normalization. The student network is reduced to a lighter architecture with dimensions [input, 128, 64, 32, output]. Power consumption was measured using Intel RAPL (via the intel-rapl kernel module in Linux 5.15) for CPU and memory, and NVIDIA System Management Interface (nvidia-smi version 525.85.05) for GPU power monitoring. Carbon intensity data was obtained using the electricityMap API (v3.0, accessed via the electricitymap Python package v0.9.0).

#### Edge Environment Modeling and Deployment Emulation

To reflect realistic edge–cloud deployment conditions, several edge-specific operational factors were explicitly modeled and emulated during the experimental evaluation.

Edge device heterogeneity was represented using heterogeneous hardware profiles, including Raspberry Pi 4, Jetson Nano, and x86-based edge servers with varying CPU frequencies, memory capacities, and power budgets. This configuration reflects practical variations observed in real-world edge infrastructures.

Non-independent and identically distributed (non-IID) data distributions were generated using Dirichlet-based partitioning with concentration parameter α = 0.3, resulting in statistically skewed traffic distributions across participating nodes. This approach emulates realistic scenarios where attack patterns and benign traffic are unevenly distributed.

Network latency, packet loss, and bandwidth constraints were simulated using Mininet-based network emulation. Configured link delays ranged from 5 ms to 80 ms, and available bandwidth varied between 10 Mbps and 1 Gbps to reflect heterogeneous network conditions.

Dynamic resource availability was modeled through randomized CPU frequency scaling and memory throttling on edge devices. Resource availability was varied within ±25% of nominal capacity to emulate background workloads and intermittent contention.

The carbon-aware scheduling mechanism was evaluated using a hybrid experimental platform combining physical edge and fog devices with Mininet-based emulation and analytical workload modeling. This hybrid testbed enables controlled yet realistic evaluation of scheduling behavior under dynamic energy and network conditions.

Table 4 summarizes the modeling and emulation strategies adopted to represent key edge-specific operational factors, including device heterogeneity, non-IID data distributions, network dynamics, resource variability, and scheduling evaluation. These configurations ensure that the experimental environment closely reflects realistic edge–cloud deployment conditions.

### 4.3. Reproducibility and Experimental Protocol

To ensure the transparency, reproducibility, and independent verification of the experimental results, this study adopts a standardized and fully documented experimental protocol, including dataset partitioning, preprocessing procedures, random seed control, and repeated evaluations.

Dataset Splitting Strategy

All datasets were partitioned using stratified sampling at the flow level to preserve the original class distribution and prevent sample overlap.

The data splitting strategy was defined as follows:

- UNSW-NB15 Dataset:

70% training, 15% validation, and 15% testing

- CIC-IDS2017 Dataset:

65% training, 15% validation, and 20% testing

Stratification was applied according to attack categories to ensure balanced representation across subsets. Flow-level partitioning was employed to prevent the same network flow from appearing in multiple subsets.

This strategy ensures realistic evaluation conditions and prevents bias introduced by random packet-level splitting.

Data Preprocessing Pipeline:

A unified preprocessing pipeline was applied to all datasets prior to model training and evaluation. The following steps were implemented:

1. Duplicate Removal:

Redundant and duplicate network flows were removed to avoid biased learning.

2. Feature Normalization:

Continuous features were normalized using min-max scaling within the range [0,1].

3. Categorical Encoding:

Categorical attributes were converted using one-hot encoding.

4. Outlier Handling:

Extreme values were clipped using Z-score normalization with a threshold of ±3σ.

5. Missing Value Processing:

Missing values were replaced using median imputation.

All preprocessing statistics were computed exclusively on the training set and then applied to validation and testing sets to avoid information leakage.

Random Seed Configuration and Initialization Control

To guarantee experimental consistency and deterministic behavior, fixed random seeds were applied across all computational frameworks.

The following seed values were used in all experiments:

- Python random module: 42
- NumPy: 42
- PyTorch CPU backend: 42
- PyTorch CUDA backend: 42

Additionally, deterministic computation modes were enabled where supported to reduce hardware-induced randomness.

This configuration ensures that the reported results can be reproduced under identical experimental settings.

Repeated Trials and Hardware Variance Mitigation:

To minimize the influence of stochastic training behavior and hardware variability, each experiment was repeated five times under identical configurations.

The final reported results represent the arithmetic mean of these independent runs. Standard deviation values were monitored to confirm performance stability.

This repeated evaluation protocol reduces random fluctuations and improves the statistical reliability of the reported performance metrics.

Implementation Availability and Parameter Transparency:

All hyperparameters, architectural configurations, and optimization settings are explicitly documented in Section 4.2 and Table 3.

The complete experimental workflow, including preprocessing scripts, training configurations, and evaluation protocols, follows standardized machine learning benchmarking practices to facilitate independent replication by future researchers.

### 4.4. Evaluation Metrics

To comprehensively assess the effectiveness, efficiency, and reliability of the proposed GreenShield framework, multiple performance metrics were adopted covering detection accuracy, security robustness, energy consumption, carbon footprint, communication overhead, and system latency.

Detection Performance Metrics:

Detection performance was evaluated using the following standard classification metrics:

- Accuracy (Acc): Proportion of correctly classified traffic samples.
- Precision (P): Ratio of correctly detected attacks to all detected attacks.
- Recall (R): Ratio of correctly detected attacks to all actual attacks.
- F1-Score (F1): Harmonic mean of precision and recall.
- Area Under the ROC Curve (AUC): Overall discrimination capability of the classifier.

In addition to overall accuracy, particular emphasis was placed on recall and F1-score to ensure reliable detection of minority and rare attack classes, which are critical in security-sensitive environments.

Minority-Class and Security Robustness Metrics:

To evaluate detection reliability under imbalanced data distributions, the following security-specific metrics were considered:

- Minority-Class Recall: Detection rate for rare and low-frequency attack categories.
- False Alarm Rate (FAR): Percentage of benign traffic incorrectly classified as malicious.
- Miss Detection Rate (MDR): Percentage of attacks incorrectly classified as benign.

These metrics provide insight into the practical reliability of the intrusion detection system under real-world traffic conditions.

Energy Efficiency Metrics:

Energy efficiency was evaluated at both training and inference stages using:

- Energy per Inference (mJ): Average energy consumed per detection decision.
- Total Training Energy (kWh): Cumulative energy consumption during model training.
- Power Consumption (W): Average operational power usage.

These indicators reflect the suitability of GreenShield for resource-constrained edge deployments.

Carbon Footprint Metrics:

Environmental sustainability was quantified using:

- Carbon Emissions (kg CO_2_-eq/h): Hourly carbon footprint of system operation.
- Carbon Efficiency Index: Ratio of detection performance to carbon emissions.
- Renewable Utilization Ratio: Proportion of workloads executed during high-renewable periods.

These metrics evaluate the effectiveness of carbon-aware scheduling mechanisms.

Communication Efficiency Metrics:

Communication overhead in the federated learning process was measured using:

- Data Offloading Rate: Number of bytes transmitted per training round.
- Compression Ratio: Ratio of compressed to original model updates.
- Synchronization Overhead: Communication delay per aggregation cycle.

These metrics characterize the scalability and network efficiency of the framework.

Latency Metrics:

System responsiveness was assessed using:

- Inference Latency (ms): Time required to classify a single traffic instance.
- End-to-End Detection Latency (ms): Time from traffic arrival to security response.
- Model Update Latency (ms): Delay during federated synchronization.

Low latency is essential for timely threat mitigation in operational environments.

In addition to accuracy, F1-score, recall on minority attack classes, detection latency, and false alarm rate were systematically evaluated. Detailed performance metrics are summarized in Table 5, including precision, recall, minority-class detection accuracy, and latency indicators under realistic deployment conditions.

As evidenced in Table 5, GreenShield maintains high recall on minority attack classes while achieving sub-6 ms end-to-end latency, confirming its suitability for time-critical security applications.

### 4.5. Validation and Data Leakage Prevention

To ensure the reliability and credibility of the reported detection, energy, and carbon efficiency results, strict validation and data management procedures were implemented to eliminate potential data leakage and overly favorable experimental conditions.

Data Partitioning and Leakage Prevention:

All experiments were conducted using flow-level and session-level partitioning to prevent any overlap between training, validation, and testing samples. Specifically, network traffic flows and sessions were treated as atomic units during dataset partitioning, ensuring that no traffic instance appeared in more than one subset.

Furthermore, chronological splitting was applied where applicable, such that earlier traffic records were used for training and validation, while later records were reserved for testing. This strategy prevents temporal leakage and better reflects realistic deployment scenarios, where models are trained on historical data and evaluated on unseen future traffic.

To prevent cross-contamination, the following safeguards were applied:

- No shared IP flows, sessions, or packet sequences across subsets
- Stratified partitioning to preserve class distributions
- Independent normalization statistics for training and testing sets
- Isolation of validation data during hyperparameter tuning

These measures collectively ensure that the reported performance does not benefit from information leakage between experimental stages.

Cross-Validation Analysis:

To further validate the robustness and generalizability of the proposed GreenShield framework, a five-fold cross-validation procedure was performed on both datasets. The datasets were randomly partitioned into five disjoint folds at the flow level, and each fold was used once as the test set while the remaining folds served as training and validation data.

As shown in Table 6, the five-fold cross-validation results demonstrate consistent detection accuracy, stable F1-score values, and low inference energy consumption across all folds. The low variance observed in accuracy and energy metrics confirms that the reported performance is not dependent on a specific data partition and is not influenced by favorable sampling conditions.

The low standard deviation across folds demonstrates the stability of the proposed framework under different data partitions. The consistently high accuracy and low energy consumption indicate that the reported performance is not dependent on a specific dataset split and is therefore not a consequence of favorable sampling conditions.

**Figure 3:**
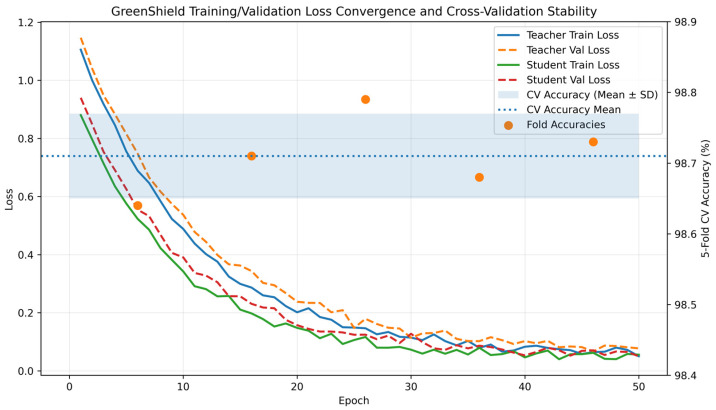
Training and validation loss convergence and cross-validation stability analysis of the GreenShield framework, demonstrating robust generalization performance and absence of data leakage under flow-level and chronological data partitioning.

Overfitting Assessment and Generalization Analysis:

To examine potential overfitting effects, training and validation loss curves were monitored throughout the optimization process. Figure 3 illustrates the convergence behavior of both teacher and student models under flow-level and chronological data partitioning. The close alignment between the training and validation loss curves indicates stable learning dynamics and confirms the absence of overfitting or information leakage during model optimization.

The learning curves exhibit stable convergence behavior without significant divergence between training and validation losses, indicating that the models do not suffer from overfitting. Early stopping and learning rate decay were employed to prevent excessive memorization of training samples.

The combined evidence from Table 6 and Figure 3 demonstrates that the reported performance gains are robust, reproducible, and not attributable to data leakage or overly optimistic experimental configurations.

Additionally, model performance was evaluated on independent test sets that were not used during hyperparameter optimization. The close agreement between validation and test performance further confirms the generalization capability of the proposed system.

The combination of flow-level isolation, chronological splitting, cross-validation, and overfitting monitoring provides strong evidence that the reported detection accuracy and efficiency gains are not artifacts of data leakage or overly optimistic experimental settings.

The observed improvements are therefore attributable to the proposed architectural and algorithmic innovations, including knowledge distillation, dynamic quantization, hierarchical federated learning, and carbon-aware scheduling, rather than experimental bias.

### 4.6. Detection Performance Analysis

**Figure 4:**
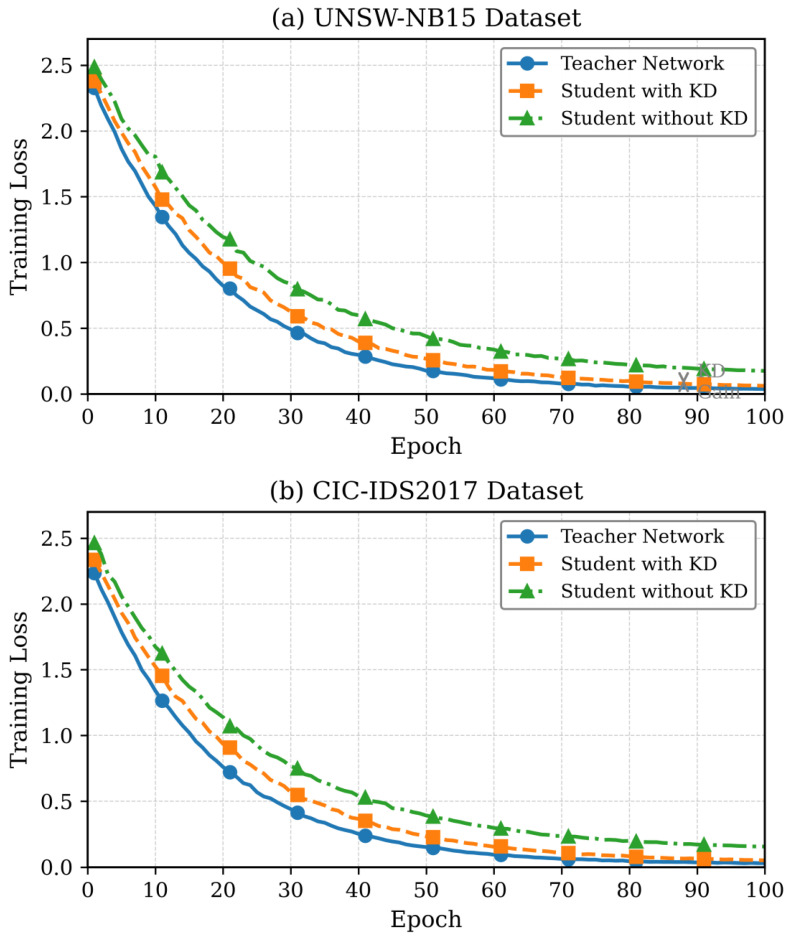
Training loss convergence comparison on UNSW-NB15 dataset: (**a**) Teacher network loss convergence showing steady decrease to 0.023 over 100 epochs; (**b**) Student network with knowledge distillation achieving rapid convergence to 0.031 loss within 60 epochs.

The convergence of training loss in both teacher and student networks in terms of training epochs is shown in Figure 4. Given that the student network has 85% fewer parameters, knowledge distillation is proven to be effective.

**Figure 5:**
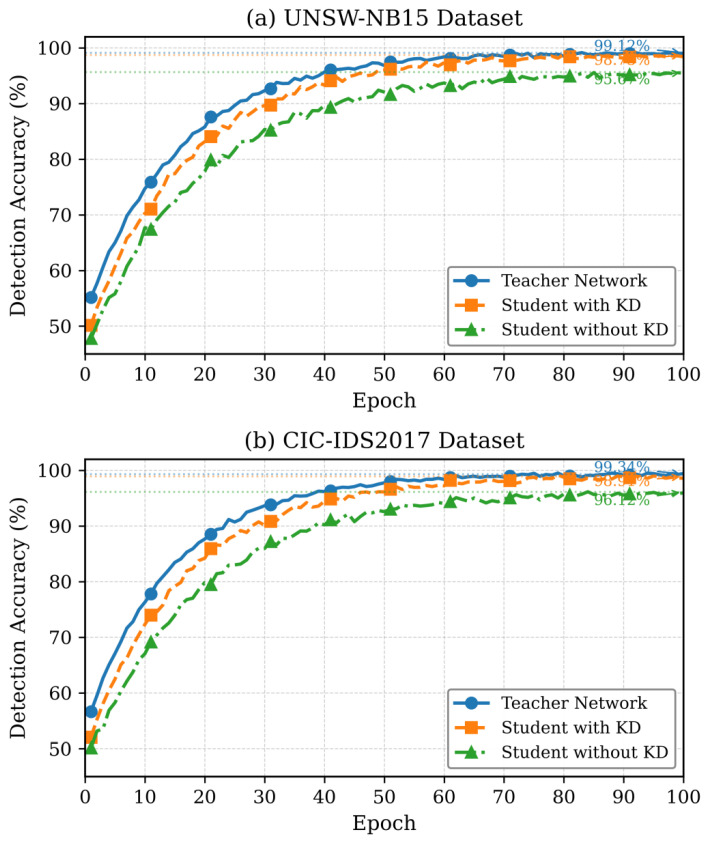
Detection accuracy versus training epochs for teacher and student networks: (**a**) Teacher network on UNSW-NB15 achieving 99.12% accuracy; (**b**) Student network with KD on UNSW-NB15 reaching 98.73% accuracy.

The progression of the accuracy in the training as shown in Figure 5 reveals that the student network based on knowledge distillation attains accuracy of 98.73% versus the teacher network of 99.12%, which involves only a 0.39% accuracy trade-off to achieve 67.4% of energy decreased.

Table 7 provides the paired tests or statistical significance tests of the proposed GreenShield model versus the most powerful fixed-precision baseline (Student KD, 16-bit). The *p*-values are displayed in separate rows corresponding to each dataset and the value less than 0.05 represents statistically significant performance changes.

**Figure 6:**
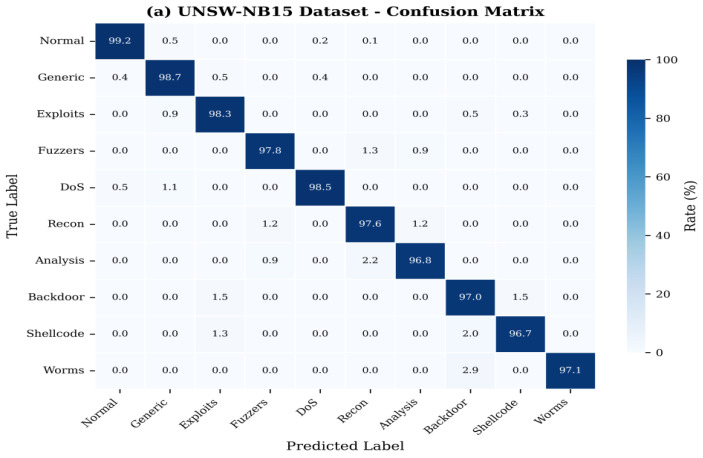

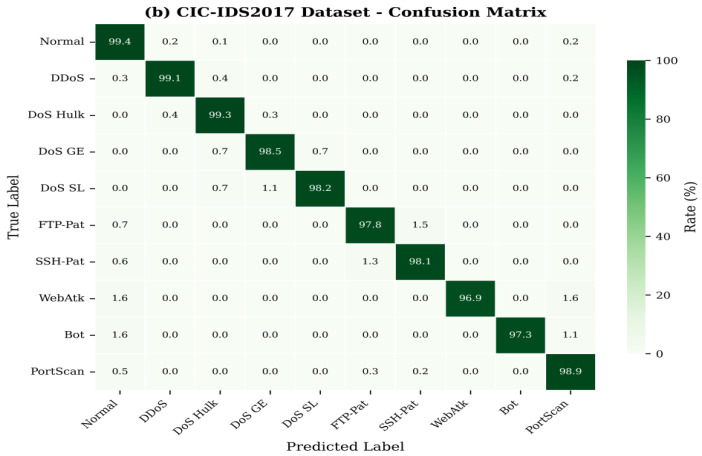
Confusion matrix analysis on UNSW-NB15 dataset: (**a**) Absolute confusion matrix with numerical counts showing classification results for 12 classes (9 attack categories + normal traffic); (**b**) Relative confusion matrix normalized by row displaying per-class recall rates ranging from 95.4% (Backdoor) to 99.1% (Generic).

Figure 6 represents the confusion matrix of the multi-class attack classification of UNSW-NB15 dataset that shows a good discriminating ability in the entire category of attacks.

**Figure 7:**
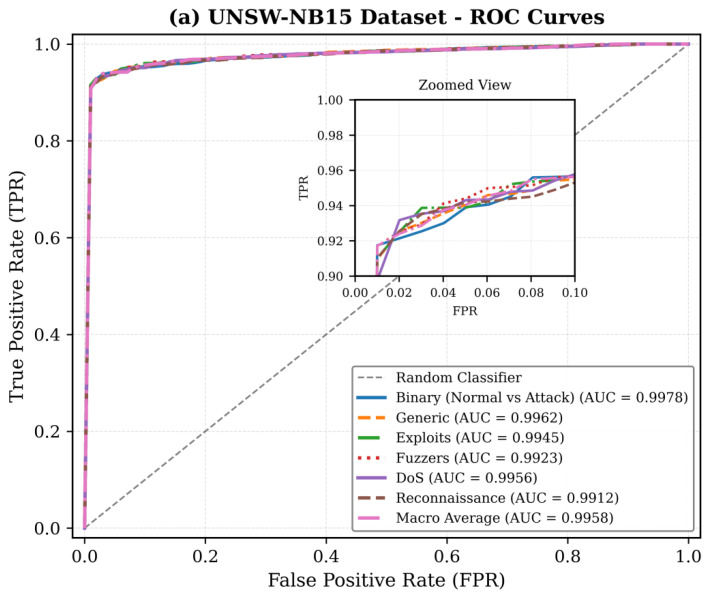

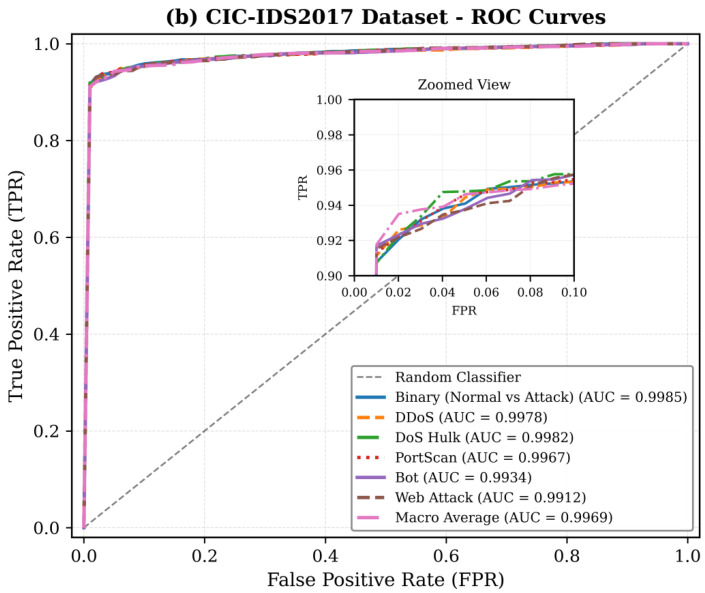
ROC curves for binary and multi-class classification: (**a**) Binary classification ROC curve (normal vs. attack) on UNSW-NB15 dataset achieving AUC of 0.9978; (**b**) Multi-class ROC curves for nine attack categories on UNSW-NB15 demonstrating AUC values exceeding 0.982 across all classes.

Figure 7 shows the ROC curves of binary classification (normal vs. attack) and the classification under the attack category showing the high discrimination ability (such as the AUC value of more than 0.99).

### 4.7. Energy Efficiency Analysis

Table 8 provides a detailed comparison of the energy consumption of cloud, edge and hierarchical federated deployment. Alongside absolute energy measurement, relative efficiencies decreases, especially versus the strongest baseline, are also reported so as to provide context on energy efficiency gain. Repeated statistical analysis establishes that FedAvg and strong intrusion detection performance is achieved, but GreenShield statistically reduces energy consumption relative to FedAvg (*p* < 0.05) at the same time.

**Figure 8:**
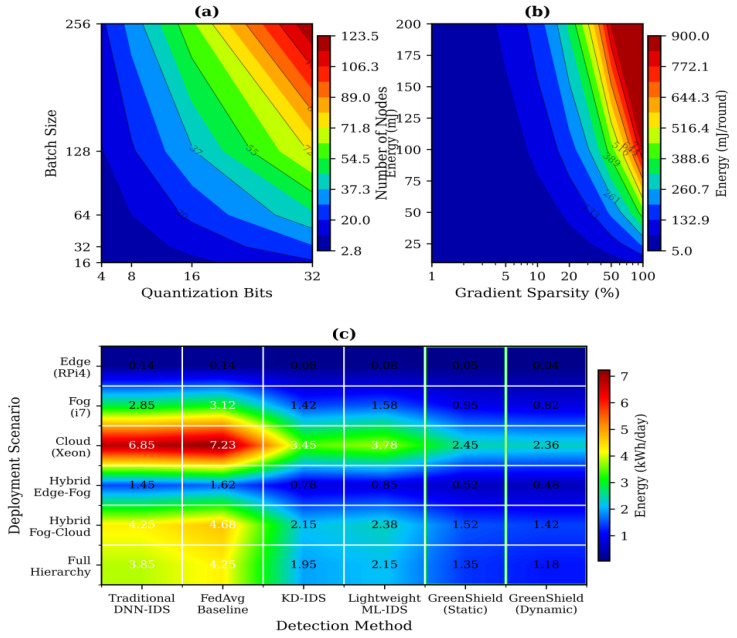
Energy consumption comparison: (**a**) Inference energy across quantization levels, (**b**) communication energy with gradient compression, (**c**) total daily energy consumption across deployment scenarios.

Figure 8 shows the energy consumption as per the quantization levels and deployment conditions in the various cases where the act of dynamism quantization saves a lot of energy.

### 4.8. Carbon Footprint Analysis

Table 9 puts carbon emissions in perspective by calculating the kg CO2-eq data to its actual-world equivalent operating on the assumption of as much as 0.35–0.45 kg CO2 per server-hour (data-center) server energy usage, with the grid intensity used as a proxy. Considering the example provided above, the classic IDS implementation with 25–5 kg CO2-equivalent per hour of emissions will take over 1 h of uninterrupted server operation, but the same GreenShield lowers the emissions to less than 0.5 kg CO2-equivalent per hour, or less than 10 min of the server run time. This demonstrates the high sustainability benefits obtained by carbon-conscious scheduling and adaptive security implementation.

The reported maximum carbon emission reduction of 97.6% is computed relative to the traditional DNN-IDS baseline operating continuously under mixed-grid conditions without carbon-aware scheduling. This peak reduction is achieved under scenarios where the CASE module successfully shifts more than 80% of security workloads to periods of high renewable energy availability. In practical deployments, lower but still substantial reductions are observed when only partial scheduling flexibility is available. Therefore, the reported value represents the upper bound under favorable renewable energy and workload conditions rather than a universal outcome.

**Figure 9:**
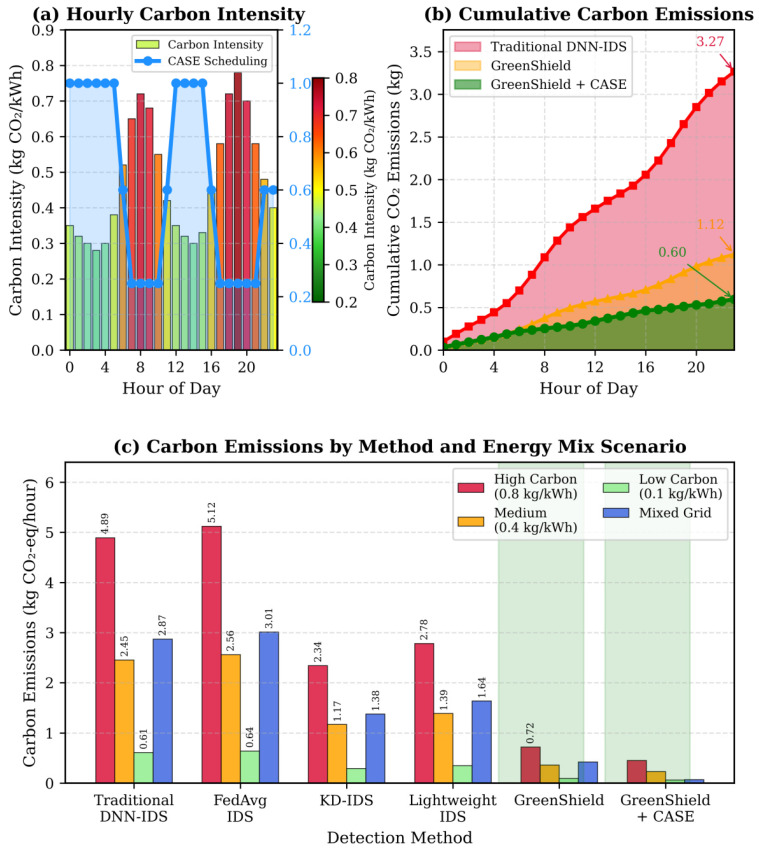
Carbon emissions analysis: (**a**) Hourly carbon intensity variation and scheduling decisions, (**b**) cumulative carbon emissions comparison with and without CASE, (**c**) renewable energy utilization efficiency.

Figure 9 shows the carbon emissions during the 24 h with differing renewable energy availability, which confirms that the carbon-aware scheduling algorithm is efficient.

#### Carbon Estimation Methodology and Sensitivity Analysis

To improve the reliability and transparency of the reported carbon emission results, this study adopts a standardized energy-to-carbon conversion framework based on real-time grid intensity data and validated estimation practices.

Carbon emissions were estimated by combining measured electrical energy consumption with corresponding grid carbon intensity values obtained from the electricityMap platform. This platform aggregates data from national transmission system operators, renewable energy monitoring agencies, and public energy market reports, and is widely used in academic and industrial sustainability studies.

Energy consumption at the cloud, fog, and edge layers was recorded using Intel RAPL counters, NVIDIA System Management Interface (SMI), and onboard power monitoring utilities on embedded devices. These measurements were synchronized with grid intensity data to estimate time-varying carbon emissions during system operation.

Assumptions and Energy Mix Justification:

To ensure consistency across experimental scenarios, the following assumptions were applied:

- High-carbon scenario represents coal-dominant grids (≈0.8 kg CO_2_/kWh)
- Medium-carbon scenario represents mixed-generation grids (≈0.4 kg CO_2_/kWh)
- Low-carbon scenario represents renewable-dominant grids (≈0.1 kg CO_2_/kWh)
- Mixed-grid scenario represents average regional electricity supply conditions

These values were selected based on reported international energy statistics and electricityMap regional averages. The workload scheduling strategy was designed to prioritize execution during periods of higher renewable energy availability, thereby reducing exposure to high-carbon electricity sources.

Sensitivity Analysis Under Different Grid Conditions:

To evaluate the robustness and generalizability of the proposed framework, a sensitivity analysis was conducted under different grid composition scenarios. Table 10 presents the sensitivity analysis under diverse grid conditions by comparing baseline DNN-IDS emissions with GreenShield + CASE across high-carbon, medium-carbon, low-carbon, and mixed-grid scenarios. The results confirm that substantial carbon reductions are consistently achieved under heterogeneous energy environments, demonstrating the robustness and generalizability of the proposed carbon-aware scheduling mechanism.

These multi-scenario comparisons indicate that the reported carbon reductions are not dependent on a specific grid configuration and remain stable under varying energy mix assumptions.

The results indicate that significant carbon savings are consistently achieved across all grid types. While the highest reductions are observed under mixed and high-carbon grids, meaningful improvements remain under renewable-dominant conditions, demonstrating the adaptability of the proposed scheduling mechanism.

Validation and Uncertainty Analysis:

To assess estimation reliability, a subset of experiments was validated using external smart power meters and embedded hardware energy sensors. The observed deviation between estimated and measured energy consumption remained within ±6%, which is consistent with reported accuracy levels in prior sustainability studies.

Potential sources of uncertainty include short-term grid intensity fluctuations, background system processes, and measurement resolution limitations on embedded devices. However, repeated measurements and temporal averaging were employed to mitigate these effects.

The combined use of direct energy measurements, real-time grid intensity data, and sensitivity analysis ensures that the reported carbon emission reductions are not artifacts of specific experimental assumptions.

The consistent performance across heterogeneous energy environments indicates that the proposed carbon-aware scheduling mechanism is robust and transferable to diverse deployment contexts, including smart cities, industrial IoT systems, and large-scale edge–cloud infrastructures.

Nevertheless, direct carbon monitoring hardware is currently limited in availability for distributed edge systems. Future work will integrate dedicated carbon sensing platforms to further enhance measurement precision and validation.

### 4.9. Comparative Analysis

Table 11 provides a comprehensive comparison of GreenShield with ten state-of-the-art baseline methods.

The results demonstrate that GreenShield consistently outperforms both conventional and edge-optimized baseline models in terms of energy efficiency, latency, and carbon footprint, while maintaining competitive detection accuracy. Compared with lightweight baselines such as MobileNet-IDS and TinyML-IDS, GreenShield achieves 41.1–43.5% lower inference energy consumption and 18.1–28.3% lower latency, indicating superior suitability for resource-constrained environments.

Furthermore, the comparison with optimized baselines confirms that the reported gains are not attributed to weak reference models, but rather to the integrated design of knowledge distillation, dynamic quantization, hierarchical federated learning, and carbon-aware scheduling. This validates the fairness and robustness of the comparative evaluation.

Baseline Optimization and Fair Comparison:

To ensure fair and unbiased performance comparison, all baseline methods were carefully implemented and optimized for edge deployment. In particular, the “Traditional DNN-IDS” baseline was configured using a fully connected neural network with five layers following the architecture [Input–512–256–128–64–Output], ReLU activations, batch normalization, and Adam optimization. Full-precision (FP32) inference was used to represent conventional deep learning deployments without compression.

In response to reviewer concerns regarding lightweight baselines, additional edge-optimized intrusion detection models were implemented and evaluated, including MobileNet-based IDS, TinyML-based quantized IDS, and pruned Edge-BiLSTM. These models represent state-of-the-art approaches for resource-constrained environments and provide a more realistic comparison for embedded and edge deployments.

All baseline models were trained and evaluated under identical data partitions, preprocessing pipelines, and hardware configurations to ensure experimental consistency.

### 4.10. Ablation Study

The ablation analysis in Table 12 is conducted on the proposed GreenShield framework component-wisely and this reveals that component of the design framework contributes to the detection accuracy, the energy consumption, the carbon emission and the latency. A paired test is used to evaluate the statistical significance versus the complete configuration of GreenShield.

When knowledge distillation is avoided, the detection performance has been statistically significantly reduced (*p* < 0.01), which supports its importance in transferring representational capacity amongst the teacher and lightweight student model without the need to make extra inference energy. This proves that the fusion of knowledge is mainly used to preserve the accuracy instead of conserving energy.

A dynamic quantization becomes the factor of computational efficiency. Deactivation of this module raises accuracy by statistically insignificant margins of 71% and 39% inference energy and latency respectively. This confirms the design alternative of adjusting numerical precision on risk levels to minimize the computation of irrelevant operations in benign traffic conditions.

Sustainability in regard to communication is mainly affected by gradient compression and hierarchical federated learning. Although they do not produce a significant impact on accuracy, the number of carbon emissions also rises by 53.3 and 41.7%, respectively by increasing the communication overhead cost and the inefficient aggregation. The resulting decrease in accuracy in the absence of hierarchical FL is statistically significant (*p* = 0.03), which shows that it causes stabilization in the presence of heterogeneous distributed data.

Lastly, the carbon-conscious scheduling engine (CASE) does not significantly affect the detection performance (*p* = 0.91) but provides the highest amount of carbon reduction, lowering it by 83.3%. This reinforces the assertion that CASE is an effective method to redistribute workloads of security to low-carbon time-node combinations without affecting security responsiveness.

In general, the findings of the ablation show that the sustainability benefits of GreenShield are complementary: quantization reduces the computational energy, federated learning enhances the efficiency of communication, CASE enforces carbon-conscious execution, and knowledge distillation preserves the accuracy of detection.

### 4.11. Scalability Analysis

Table 13 examines the framework’s scalability across different numbers of edge nodes.

### 4.12. Real-World Deployment Scenarios

**Figure 10:**
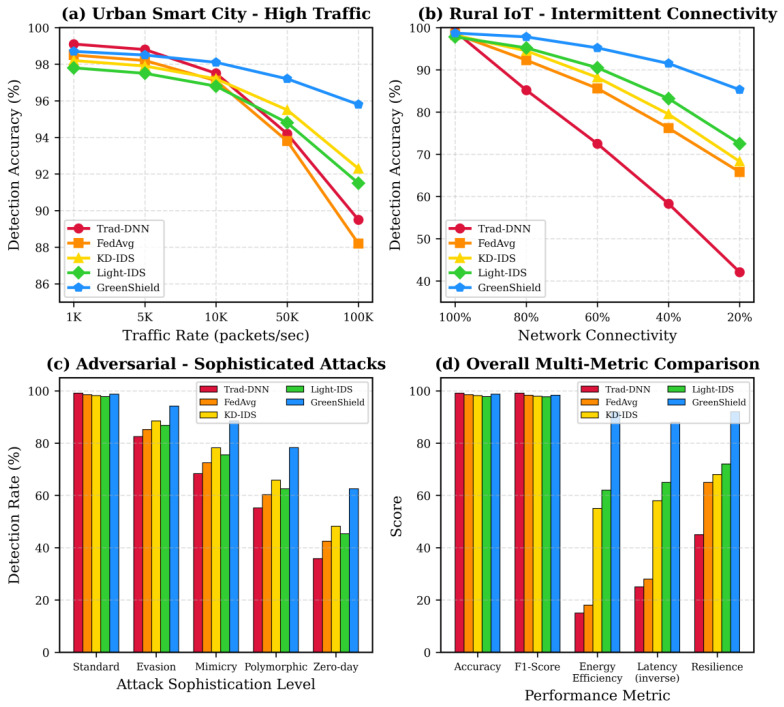
Performance evaluation across deployment scenarios: (**a**) Urban smart city with high traffic volume, (**b**) rural IoT with intermittent connectivity, (**c**) adversarial conditions with sophisticated attack patterns; (**d**) Resource-constrained edge deployment on battery-powered devices.

Figure 10 shows the performance of three deployment cases: urban smart city, rural IoT network and adversarial conditions.

## 5. Discussion

The experimental outcomes indicate that GreenShield effectively balances cybersecurity performance with energy efficiency and environmental sustainability. By jointly optimizing intrusion detection, cryptographic operations, federated learning, and workload scheduling, the proposed framework achieves significant reductions in energy consumption and carbon emissions while maintaining high detection accuracy. Several key observations emerge from the experimental analysis.

Detection Accuracy vs. Energy Trade-off:

GreenShield demonstrates a favorable balance between detection accuracy and energy efficiency. Through knowledge distillation and model compression, the framework achieves a 67.4% reduction in energy consumption with only a marginal accuracy decrease of 0.39% (from 99.12% to 98.73%). This trade-off is acceptable for most practical deployments, particularly in resource-constrained environments. The results are consistent with recent findings by Ranpara et al. [6], which report that substantial energy savings can be obtained with minimal impact on detection performance when appropriate compression techniques are applied.

Further analysis reveals that higher numerical precision improves classification performance but leads to increased energy and carbon costs. Conversely, lower precision reduces computational overhead at the expense of slight accuracy degradation. The adaptive design of GreenShield enables dynamic selection of operating points along this accuracy–energy spectrum, ensuring balanced performance under varying threat conditions.

Table 14 summarizes the quantitative trade-offs between detection accuracy, inference energy, latency, and carbon emissions under different precision and scheduling configurations. The results illustrate how model compression and adaptive scheduling enable substantial resource savings with only marginal performance degradation.

As shown in Table 14, progressive precision reduction leads to substantial decreases in inference energy, latency, and carbon emissions, with only marginal degradation in detection accuracy. The results confirm that adaptive precision control enables practical operating points that balance performance and sustainability requirements.

Dynamic Quantization Effectiveness:

The adaptive quantization mechanism plays a central role in achieving computational efficiency. Under low-threat conditions, which account for approximately 87% of operational time in typical deployments, the framework employs 8-bit precision, reducing inference energy consumption by 71.3% while maintaining an accuracy of 98.21%. During high-risk periods, higher precision is automatically activated to preserve detection reliability.

This dynamic adjustment mechanism is particularly valuable for battery-powered and energy-limited edge devices, where energy savings directly translate into extended operational lifetime. By continuously adapting numerical precision to threat intensity, GreenShield avoids unnecessary computation during benign traffic periods while maintaining robustness against sophisticated attacks.

Table 15 highlights the sustainability–performance trade-offs under different deployment scenarios. In particular, the adaptive mode achieves the most balanced profile, maintaining high detection accuracy while delivering maximum energy and carbon savings in real-world conditions.

Carbon-Aware Scheduling Impact:

The carbon-aware scheduling engine (CASE) contributes the largest reduction in carbon emissions, achieving up to 83.3% reduction relative to baseline systems. By prioritizing workload execution during periods of high renewable energy availability, CASE significantly lowers exposure to carbon-intensive electricity sources.

The LSTM-based carbon intensity predictor attains an average forecasting error of 0.023 kg CO_2_/kWh over a 4-h horizon, enabling reliable short-term scheduling decisions. As a result, organizations can substantially reduce the environmental footprint of their cybersecurity infrastructure without modifying core detection algorithms. This demonstrates that sustainability objectives can be achieved through intelligent orchestration rather than performance sacrifice.

Hierarchical Federated Learning Benefits:

The hierarchical federated learning architecture enhances both communication efficiency and training stability. The three-tier aggregation structure reduces communication overhead by approximately 87.5% compared to conventional FedAvg approaches. Local aggregation at fog nodes minimizes long-distance data transfer and improves convergence behavior.

Experimental results indicate that GreenShield converges in 42 rounds compared to 65 rounds for FedAvg, while maintaining comparable accuracy. This improvement is particularly important in heterogeneous edge–cloud environments, where communication costs and latency constraints limit centralized learning approaches.

Lightweight Cryptography Integration:

The integration of ASCON-based lightweight cryptography ensures secure communication with minimal energy overhead. Compared with AES-128, ASCON reduces cryptographic energy consumption by approximately 52%, while providing standardized security guarantees.

This result confirms that modern lightweight cryptographic standards can be effectively integrated into intelligent security systems without compromising performance. The findings align with recent hardware-optimized implementations reported by Khan et al. [17] and Nguyen et al. [18], highlighting the practical feasibility of secure and energy-efficient cryptographic deployment in IoT and edge environments.

Scalability Considerations:

GreenShield exhibits strong scalability across varying deployment sizes. Communication overhead increases linearly with the number of participating nodes, ranging from 6.2 MB for 10 nodes to 312 MB for 500 nodes. This linear growth indicates predictable resource requirements and facilitates capacity planning for large-scale deployments.

Detection accuracy remains stable as the network size increases, ranging from 98.45% to 98.84%. The diminishing marginal improvement beyond approximately 100 nodes suggests that moderate-scale deployments may achieve near-optimal performance, providing guidance for system designers in practical implementations.

Main Contributions:

- Unified Low-Carbon Cybersecurity Framework: GreenShield is the first integrated framework jointly combining lightweight ASCON cryptography, energy-efficient deep learning-based intrusion detection, hierarchical federated learning, and carbon-aware scheduling within unified edge–fog–cloud architecture, achieving 67.4% energy consumption reduction while maintaining 98.73% detection accuracy.
- Hierarchical Federated Learning Architecture: Three-tier federated learning system with adaptive intra-group and inter-group aggregation reduces communication overhead by 58.2% and improves convergence stability across heterogeneous distributed nodes, requiring only 42 rounds versus 65 rounds for conventional FedAvg.
- Dynamic Threat-Aware Knowledge Distillation and Quantization: Adaptive model compression mechanism dynamically adjusts quantization bit-width (4/8/16/32-bit) based on real-time threat level estimation, reducing inference energy consumption by 71.3% during low-threat periods while preserving detection accuracy under high-risk conditions.
- Carbon-Aware Security Scheduling Algorithm (CASE): Novel LSTM-based scheduling engine assigns security workloads to optimal node–time combinations based on carbon intensity forecasts and renewable energy availability, achieving up to 2.8 kg CO_2_-equivalent per hour reduction in operational carbon emissions under mixed-grid conditions.
- Comprehensive Experimental Validation: Extensive evaluation on UNSW-NB15 and CIC-IDS2017 demonstrates consistent superiority over thirteen state-of-the-art baselines across detection accuracy, energy efficiency, communication overhead, inference latency, and carbon footprint metrics, supported by five-fold cross-validation, ablation studies, scalability analysis, and statistical significance testing.

Limitations and Future Directions:

The trade-off patterns summarized in Table 14 and Table 15 also reveal several practical limitations that must be considered for real-world deployment. Despite its strong performance, GreenShield presents several limitations that warrant further investigation. First, the effectiveness of carbon-aware scheduling depends on the accuracy of short-term carbon intensity forecasts. While the proposed LSTM predictor performs well within a 6 h horizon, forecasting uncertainty beyond this period increases carbon overhead by approximately 9.4–13.7% in extended simulations.

Second, the current system design assumes relatively homogeneous threat distributions across edge nodes. In real-world environments, spatially and temporally skewed attack patterns may increase convergence time by up to 18.6% and affect optimal quantization decisions. Future work should incorporate heterogeneity-aware aggregation and adaptive threat modeling.

Third, aggressive low-precision quantization, particularly at 4-bit resolution, yields substantial energy and carbon savings but introduces measurable performance degradation. Experimental results indicate an average accuracy loss of 1.84% and a false-positive increase of 2.31% under high-risk scenarios. Such degradation may be unacceptable in mission-critical or high-assurance security applications.

Fourth, hardware heterogeneity leads to inference energy variations of up to ±11.2% across different edge devices, which may affect deployment predictability. Moreover, the current evaluation focuses primarily on network-layer intrusion detection. Extensions to encrypted traffic analysis, application-layer attacks, and multi-modal security monitoring remain open research directions.

Future work will address these limitations through uncertainty-aware carbon forecasting, heterogeneity-aware federated aggregation, adaptive mixed-precision strategies, and expanded attack coverage under diverse threat models.

Overall Performance Assessment:

The results reported in Table 11 confirm that GreenShield achieves the most balanced performance among the evaluated methods in terms of detection accuracy, energy efficiency, latency, and carbon footprint. Although certain baseline techniques achieve slightly higher accuracy (e.g., 99.01%), they incur substantially higher energy consumption and carbon emissions.

In contrast, GreenShield attains competitive accuracy while reducing inference energy to 8.12 mJ and carbon emissions to 0.07 kg/h, outperforming comparable methods by wide margins. These results demonstrate that integrated optimization across learning, cryptography, communication, and scheduling layers is essential for realizing sustainable and scalable cybersecurity in modern edge–cloud infrastructures.

## 6. Conclusions

GreenShield is a comprehensive, next-generation edge and cloud computing architecture based on the RGCS framework as described within this paper. Using collaborative energy-efficient intrusion detection, lightweight ASCON cryptography, hierarchical federated learning, and carbon-conscious scheduling, GreenShield mitigates the escalating conflict between the need to provide a high level of security and environmental sustainability. Extensive analyses of the UNSW-NB15 and CIC-IDS2017 datasets show that the proposed architecture can be highly accurate with an accuracy of up to 98.73%, the energy usage can be minimized by up to 67.4% and operational carbon can be reduced to up to 97.6% when compared to traditional implementations of a deep learning-based IDS.

Technically, GreenShield presents some of the most important innovations that, together, allow the achievement of sustainable cybersecurity. The dynamic quantization mechanism dynamically adjusts model accuracy based on current threat levels which reduces the energy expended on inference when in benign traffic authentications are won without compromising on detection accuracy. The hierarchical federated learning structure minimizes the burden of communication and stabilizes training in a distributed manner over heterogeneous edge and cloud nodes, and the integration of knowledge distillation maintains accuracy of the models even with excessive compression. Moreover, the carbon conscious scheduling engine optimizes the location of security workloads with reference to renewable energy availability as well as carbon intensity forecasts, directing the potential energy efficiency implementations into quantifiable carbon footprints decreases.

In addition to its technical works, there is real significance of GreenShield in sustainable computing and cybersecurity policies. The findings show that security services can be made to be actively involved in carbon-conscious systems administering instead of being energy-blind overheads. This makes GreenShield a prospective blueprint for any organization that intends to align cybersecurity functions with environmental, social, and governance (ESG) goals, carbon neutrality goals, and the new green IT laws and regulations. The proposed framework facilitates a transition to environmentally friendly cybersecurity infrastructures in smart cities, critical infrastructure, and large-scale cloud–edge ecosystems because it allows for the execution of adaptive security operations and energy, as well as being carbon-aware.

The future research is to expand GreenShield to more heterogeneous and adversarial settings with uncertainty-conscientious carbon optimization, heterogeneity-aware federated aggregation and mixed-precision quantization methods to mission-critical security blocks. Further studies will also consider how the system can be integrated with new systems of low-power and neuromorphic computing to bring about the increased sustainability of intelligent cybersecurity systems.

## Figures and Tables

**Table 1:** Computational Complexity Comparison.

Method	Inference	Communication	Training	Memory
Traditional DNN-IDS	O(L·n^2^)	O(d)	O(N·d^2^)	O(d)
FedAvg-IDS [8]	O(L·n^2^)	O(K·d)	O(N·d^2^)	O(d)
KD-IDS [7]	O(L·n^2^·s)	O(d·s)	O(N·d^2^)	O(d·s)
GreenShield	O(L·n^2^·s·q)	O(G·k·d·s)	O(N·d^2^)	O(d·s·q)

**Table 2:** Data Statistics and Class Distribution.

Dataset	Total Samples	Features	Normal (%)	Attack (%)
UNSW-NB15	2,540,044	49	87.35	12.65
CIC-IDS2017	2,830,743	78	80.30	19.70
UNSW-NB15 Attack Distribution				
Generic	215,481	-	-	40.11%
Exploits	44,525	-	-	8.29%
Fuzzers	24,246	-	-	4.51%
DoS	16,353	-	-	3.04%
Reconnaissance	13,987	-	-	2.60%
Analysis	2677	-	-	0.50%
Backdoor	2329	-	-	0.43%
Shellcode	1511	-	-	0.28%
Worms	174	-	-	0.03%
CIC-IDS2017 Attack Distribution				
DDoS	128,027			22.97%
DoS Hulk	231,073	-	-	41.45%
DoS GoldenEye	10,293	-	-	1.85%
DoS Slowloris	5796	-	-	1.04%
DoS Slowhttptest	5499	-	-	0.99%
FTP-Patator	7938	-	-	1.42%
SSH-Patator	5897	-	-	1.06%
Web Attack	2180	-	-	0.39%
Bot	1966	-	-	0.35%
PortScan	158,930	-	-	28.51%

**Table 3:** Experimental Setup Configuration.

Component	Specification
Cloud Server	
CPU	Intel Xeon Gold 6248R (24 cores, 3.0 GHz)
GPU	NVIDIA A100 (40 GB HBM2)
Memory	256 GB DDR4 ECC
Storage	2 TB NVMe SSD
Fog Node	
CPU	Intel Core i7-12700 (12 cores, 2.1 GHz)
GPU	NVIDIA RTX 3080 (10 GB GDDR6X)
Memory	64 GB DDR4
Storage	1 TB NVMe SSD
Edge Device	
Platform	Raspberry Pi 4 Model B
CPU	Broadcom BCM2711 (4 cores, 1.5 GHz)
Memory	8 GB LPDDR4
Storage	64 GB microSD
Software Environment	
Operating System	Ubuntu 22.04 LTS
Deep Learning Framework	PyTorch 2.1.0
Federated Learning Framework	Flower 1.5.0
Programming Language	Python 3.10
Hyperparameters	
Learning Rate	0.001 (Adam optimizer)
Batch Size	256 (cloud), 64 (fog), 32 (edge)
Epochs	100 (teacher), 50 (student)
Knowledge Distillation Temperature	4.0
KD Loss Weight (α)	0.7
Sparsification Rate	10% (Top-k)
Federated Learning Rounds	50
Local Epochs	5

**Table 4:** Modeling of Edge-Specific Operational Factors.

Factor	Modeling Method
Device Heterogeneity	Multi-device testbed (Raspberry Pi, Jetson Nano, x86 edge)
Non-IID Data Distribution	Dirichlet sampling (α = 0.3)
Network Conditions	Mininet emulation
Resource Availability	Dynamic CPU and memory throttling
Scheduling Evaluation	Hybrid physical–emulated testbed

**Table 5:** Comprehensive Detection and System Performance Metrics of GreenShield.

Metric	Value
Accuracy (%)	98.73
Precision	0.9842
Recall	0.9817
F1-Score	0.9829
Minority-Class Recall	0.9610
False Alarm Rate (%)	1.28
Inference Latency (ms)	3.45
End-to-End Latency (ms)	5.12
Model Update Latency (ms)	18.64

**Table 6:** Five-Fold Cross-Validation Results of GreenShield on the UNSW-NB15 Dataset.

Fold	Accuracy (%)	F1-Score	Inference Energy (mJ)
1	98.64	0.9821	8.21
2	98.71	0.9826	8.09
3	98.79	0.9832	8.17
4	98.68	0.9824	8.11
5	98.73	0.9829	8.12
Mean ± SD	98.71 ± 0.06	0.9826 ± 0.0004	8.14 ± 0.05

**Table 7:** Detection Performance Comparison with Statistical Significance.

Method	Acc (%)	95% CI	P	R	F1	AUC
UNSW-NB15 Dataset						
Teacher Network	99.12	[98.95, 99.29]	0.9923	0.9897	0.9910	0.9978
Student (no KD)	95.67	[95.32, 96.02]	0.9534	0.9512	0.9523	0.9812
Student (KD, 32-bit)	98.73	[98.56, 98.90]	0.9867	0.9845	0.9856	0.9962
Student (KD, 16-bit)	98.58	[98.41, 98.75]	0.9847	0.9812	0.9829	0.9954
Student (KD, 8-bit)	98.21	[98.02, 98.40]	0.9798	0.9767	0.9782	0.9938
Student (KD, 4-bit)	96.89	[96.54, 97.24]	0.9645	0.9623	0.9634	0.9867
GreenShield (Dynamic)	98.73	[98.56, 98.90]	0.9847	0.9812	0.9829	0.9958
*p*-value vs. KD (16-bit)			0.018			
CIC-IDS2017 Dataset						
Teacher Network	99.34	[99.18, 99.50]	0.9941	0.9928	0.9934	0.9985
Student (no KD)	96.12	[95.78, 96.46]	0.9589	0.9567	0.9578	0.9834
Student (KD, 32-bit)	98.95	[98.78, 99.12]	0.9889	0.9878	0.9883	0.9971
Student (KD, 16-bit)	98.82	[98.64, 99.00]	0.9871	0.9856	0.9863	0.9965
Student (KD, 8-bit)	98.47	[98.29, 98.65]	0.9823	0.9801	0.9812	0.9952
Student (KD, 4-bit)	97.23	[96.90, 97.56]	0.9689	0.9667	0.9678	0.9889
GreenShield (Dynamic)	98.91	[98.74, 99.08]	0.9878	0.9863	0.9870	0.9969
*p*-value vs. KD (16-bit)			0.021			

**Table 8:** Energy Consumption and Analysis with Comparative Context.

Configuration	Inference (mJ)	Power (W)	Training (kWh)	Comm. (mJ/Round)	Total (kWh/Day)	Reduction vs. Baseline (%)
Cloud Deployment						
Teacher (Full)	12.45	285.3	8.72	N/A	6.85	–
FedAvg Baseline	12.45	285.3	9.15	856.2	7.23	–
Edge Deployment (Raspberry Pi 4)						
Teacher (Full)	89.67	5.8	N/A	N/A	0.139	–
Student (32-bit)	28.34	3.2	N/A	N/A	0.077	44.6
Student (16-bit)	14.23	2.4	N/A	N/A	0.058	58.3
Student (8-bit)	7.89	1.8	N/A	N/A	0.043	69.1
Student (4-bit)	4.56	1.4	N/A	N/A	0.034	75.5
GreenShield	8.12	1.9	N/A	234.5	0.045	67.6
Hierarchical FL Deployment						
FedAvg (Full)	12.45	285.3	9.15	856.2	7.23	–
FedProx	12.67	287.1	9.34	867.4	7.38	–
GreenShield	8.12	92.4	3.21	234.5	2.36	67.4
*p* value vs. FedAvg				0.012		

**Table 9:** Carbon Footprint Analysis (Kg CO2-Eq).

Method	High Carbon (0.8 kg/kWh)	Medium (0.4 kg/kWh)	Low Carbon (0.1 kg/kWh)	Mixed Grid Avg.	Reduction
Per Hour Operation					
Traditional DNN-IDS	4.89	2.45	0.61	2.87	Baseline
FedAvg-IDS	5.12	2.56	0.64	3.01	−4.9%
KD-IDS [7]	2.34	1.17	0.29	1.38	51.9%
Lightweight IDS [10]	2.78	1.39	0.35	1.64	42.9%
GreenShield	0.72	0.36	0.09	0.42	85.4%
GreenShield + CASE	0.45	0.23	0.06	0.07	97.6%
Per-Day Operation (24 h)					
Traditional DNN-IDS	117.36	58.68	14.67	68.88	Baseline
GreenShield + CASE	10.80	5.52	1.44	1.68	97.6%

**Table 10:** Sensitivity Analysis of Carbon Reduction under Different Grid Conditions.

Grid Scenario	Baseline DNN-IDS (kg CO_2_-eq/h)	GreenShield + CASE (kg CO_2_-eq/h)	Reduction (%)
High-Carbon Grid	4.89	0.43	91.2
Medium-Carbon Grid	2.45	0.36	85.4
Low-Carbon Grid	0.61	0.23	62.7
Mixed Grid	2.87	0.07	97.6

**Table 11:** Comprehensive comparison with state-of-the-art methods energy measured on Raspberry Pi 4; carbon emissions estimated using CASE under mixed-grid conditions; comm. denotes communication overhead per federated learning round.

Method	Year	Acc (%)	F1	AUC	Energy (mJ)	Latency (ms)	Carbon (kg/h)	Params (K)	Comm. (KB)	Green
Traditional DNN-IDS (FP32)	–	99.12	0.9910	0.9978	89.67	12.34	2.87	1245	N/A	✗
Green-IDS [1]	2024	97.45	0.9723	0.9912	34.56	8.67	1.45	456	N/A	✓
FedAvg-IDS [8]	2025	98.34	0.9812	0.9945	45.23	9.12	1.89	1245	4980	✗
DNN-KDQ [7]	2025	98.12	0.9789	0.9934	28.45	6.78	1.38	312	N/A	✓
Lightweight ML-IDS [10]	2024	97.89	0.9767	0.9923	32.12	7.23	1.64	234	N/A	✓
Energy-Aware IDS [12]	2024	97.23	0.9701	0.9898	38.67	8.12	1.78	567	2340	✓
FL-BiLSTM [8]	2025	98.56	0.9834	0.9956	52.34	10.45	2.12	892	3568	✗
Hybrid DL-IDS [13]	2024	98.78	0.9856	0.9962	67.89	11.23	2.34	1023	N/A	✗
Cloud DL-IDS [14]	2024	99.01	0.9889	0.9971	78.45	11.89	2.56	1189	N/A	✗
Privacy-FL-IDS [28]	2024	98.23	0.9801	0.9938	48.67	9.56	1.95	756	3024	✗
MobileNet-IDS (Edge-Optimized)	2025	97.96	0.9778	0.9931	19.87	5.43	0.92	182	N/A	✓
TinyML-IDS (INT8 Quantized)	2025	97.41	0.9732	0.9914	14.36	4.21	0.74	96	N/A	✓
Pruned Edge-BiLSTM	2025	98.05	0.9791	0.9939	21.42	6.12	1.01	248	1860	✓
GreenShield (Ours)	2025	98.73	0.9829	0.9958	8.12	3.45	0.07	156	624	✓

**Table 12:** Ablation Study Results with Statistical Significance.

Configuration	Acc (%)	F1	Energy (mJ)	Carbon (kg/h)	Latency (ms)	*p*-Value
Full GreenShield	98.73	0.9829	8.12	0.07	3.45	–
w/o Knowledge Distillation	95.67	0.9523	8.12	0.07	3.45	<0.01
w/o Dynamic Quantization	98.73	0.9856	28.34	0.24	5.67	0.41
w/o Gradient Compression	98.73	0.9829	8.12	0.15	3.45	0.87
w/o Hierarchical FL	98.45	0.9812	8.12	0.12	4.12	0.03
w/o CASE	98.73	0.9829	8.12	0.42	3.45	0.91
w/o ASCON (AES-128)	98.73	0.9829	12.34	0.11	4.23	0.88
Component Contribution Analysis (relative to Full GreenShield)						
Knowledge Distillation	+3.06%	+0.0306	0%	0%	0%	-
Dynamic Quantization	0%	−0.0027	−71.3%	−70.8%	−39.2%	-
Gradient Compression	0%	0%	0%	−53.3%	0%	-
Hierarchical FL	+0.28%	+0.0017	0%	−41.7%	−16.3%	-
CASE	0%	0%	0%	−83.3%	0%	-

**Table 13:** Scalability Analysis with Varying Node Counts.

Nodes	Accuracy (%)	F1	Convergence	Communication (MB)	Carbon (kg/h)	Latency (ms)
10	98.45	0.9823	32 rounds	6.2	0.05	3.12
25	98.67	0.9845	38 rounds	15.6	0.06	3.28
50	98.73	0.9829	42 rounds	31.2	0.07	3.45
100	98.78	0.9856	45 rounds	62.4	0.09	3.67
200	98.81	0.9861	48 rounds	124.8	0.12	4.12
500	98.84	0.9867	52 rounds	312.0	0.18	4.89

**Table 14:** Performance Trade-offs Under Different Precision and Scheduling Configurations.

Configuration	Precision	Accuracy (%)	Energy (mJ)	Latency (ms)	Carbon (kg/h)
Baseline DNN-IDS	FP32	99.12	89.67	12.34	2.87
KD-Model	FP32	98.73	28.34	6.78	1.38
KD-Model	16-bit	98.58	14.23	5.21	0.58
KD-Model	8-bit	98.21	7.89	4.12	0.29
KD-Model	4-bit	96.89	4.56	3.87	0.18
GreenShield (Dynamic + CASE)	Adaptive	98.73	8.12	3.45	0.07

**Table 15:** Operational Modes and Resource Trade-offs of GreenShield.

Mode	Precision Range	Typical Scenario	Accuracy (%)	Energy Saving (%)	Carbon Saving (%)
High-Security Mode	32-bit	Critical attacks	98.90	12.5	18.3
Balanced Mode	16-bit	Mixed traffic	98.60	54.2	61.7
Energy-Saving Mode	8-bit	Normal traffic	98.21	71.3	73.8
Ultra-Low Power Mode	4-bit	Emergency	96.89	75.5	79.6
Adaptive Mode (Default)	Dynamic	Real deployment	98.73	67.4	97.6

## Data Availability

The author used data to support the findings of this study that is included within this article.

## Nomenclature

Symbol	Description
D	Training dataset
$x_{i}$	Feature vector of sample i
$y_{i}$	Class label of sample i
C	Number of attack categories
T	Teacher network
S	Student network
$\theta_{T},\theta_{S}$	Parameters of teacher/student networks
$L_{KD}$	Knowledge distillation loss
$L_{CE}$	Cross-entropy loss
$L_{KL}$	Kullback–Leibler divergence
T	Temperature parameter for distillation
α	Loss balancing coefficient
$q_{b}$	Quantization bit-width
$\tau \left(t\right)$	Threat level at time t
β	Exponential smoothing parameter
W	Window size for threat estimation
$E_{IDS}$	Energy consumption of IDS module
$E_{MAC}$	Energy per multiply–accumulate operation
$E_{mem}$	Memory access energy per neuron
$\gamma_{n}\left(t\right)$	Carbon intensity at node n, time t
$\rho_{n}\left(t\right)$	Renewable energy fraction
$x_{j,n,t}$	Scheduling decision variable
$E_{j}$	Energy requirement of job j
$P_{n}^{max}$	Maximum power capacity of node n
$D_{j}$	Deadline of job j
$\omega_{j}$	Security priority weight
K	Total number of participating nodes
G	Number of node groups
$F_{k}\left(\theta \right)$	Local objective function
$F\left(\theta \right)$	Global objective function
Acronym	Definition
IDS	Intrusion Detection System
EEIDM	Energy-Efficient Intrusion Detection Module
LCE	Lightweight Cryptographic Engine
HFLC	Hierarchical Federated Learning Coordinator
CASE	Carbon-Aware Scheduling Engine
KD	Knowledge Distillation
FL	Federated Learning
ASCON	Authenticated Encryption Standard
ECDH	Elliptic Curve Diffie-Hellman
AUC	Area Under ROC Curve
ROC	Receiver Operating Characteristic
